# Accumbal acetylcholine signals associative salience

**DOI:** 10.1101/2025.01.06.631529

**Published:** 2025-01-06

**Authors:** Zhewei Zhang, Kauê Machado Costa, Yizhou Zhuo, Guochuan Li, Yulong Li, Geoffrey Schoenbaum

**Affiliations:** 1 National Institute on Drug Abuse Intramural Research Program, National Institutes of Health, Baltimore, MD, 21224, USA; 2 State Key Laboratory of Membrane Biology, School of Life Sciences, Peking University, Beijing 100871, China; 3 PKU-IDG/McGovern Institute for Brain Research, Beijing, 100871, China; 4 Peking-Tsinghua Center for Life Sciences, New Cornerstone Science Laboratory, Academy for Advanced Interdisciplinary Studies, Peking University, Beijing, 100871, China; 5 Chinese Institute for Brain Research, Beijing, 102206, China; 6 Institute of Molecular Physiology, Shenzhen Bay Laboratory, Shenzhen, Guangdong, 518055, China;; 7 National Biomedical Imaging Center, Peking University, Beijing, 100871, China

**Keywords:** acetylcholine, salience, dopamine, nucleus accumbens core, fiber photometry

## Abstract

Learning in dynamic environments requires animals to not only associate cues with outcomes but also to determine cue salience, which modulates how quickly related associations are updated. While dopamine (DA) in the nucleus accumbens core (NAcc) has been implicated in learning associations, the mechanisms of salience are less understood. Here, we tested the hypothesis that acetylcholine (ACh) in the NAcc encodes cue salience. We conducted four odor discrimination experiments in rats while simultaneously measuring accumbal ACh and DA. We found that ACh developed characteristic dips to cues over learning before DA signals differentiated cues by value, with these dips persisting through value decreases and developing faster during meta-learning. The dips reflected the cue salience across learning stages and tasks, as predicted by a hybrid attentional associative learning model that integrated principles from the Mackintosh and Pearce-Hall models, suggesting that accumbal ACh signals encode salience and potentially gate the learning process.

## Introduction:

Learning is critical for survival in dynamic natural environments, where numerous changes occur constantly but certain cues can reliably predict the presence of food or the approach of predators. Animals not only learn the associations between these cues and their outcomes but also learn to attribute salience to cues^1–3^. Cues with higher salience receive more attention and are more likely to be associated with relevant outcomes. These two processes are intertwined. Learning about the outcome that follows a cue influences its salience, and the salience of a cue, in turn, affects the learning rate for subsequent associations involving that cue.

Attentional associative learning models have formalized these learning processes^4–7^. Two influential models, the Mackintosh^3,4^ and Pearce-Hall models^7,8^, emphasize different aspects of salience dynamics. Mackintosh proposed that a stimulus gains salience if it reliably predicts the outcome^9,10^. This model with predictiveness-driven salience explains various phenomena, such as the observation that previously blocked cues or dimensions are slower to enter into novel associations^11,12^. In contrast, the Pearce-Hall model posits that salience is assigned to cues paired with uncertain outcomes^13,14^. This model with uncertainty-driven salience accounts for phenomena like latent inhibition^15,16^, where pre-exposure to a cue impairs subsequent learning about associations between that cue and other events. Accumulating evidence suggests that salience is affected by both predictiveness and uncertainty in certain contexts, which has led to the proposal of hybrid models^6,17^ that integrate both the predictiveness-driven and uncertainty-driven salience components. Le Pelley (2004)^17^ and Esber and Haselgrove (2011)^6^ defined and integrated these two components differently to determine a cue’s ultimate total salience, explaining a broader range of experimental findings.

Among neuromodulatory systems, cholinergic and dopaminergic neurons, which release acetylcholine (ACh) and dopamine (DA), respectively, are particularly important in learning^18–20^. These two systems interact closely^21–23^, and disruptions in either ACh^24–26^ and DA^27–29^ signals have been shown to impair various learning behaviors. Moreover, cues associated with rewards induce different phasic changes in the release of these striatal neuromodulators^30–32^. Specifically, phasic changes in DA are thought to signal reward prediction errors^30,31^ (RPE), discrepancies between expected and actual reward outcomes. With concurrent phasic pauses in ACh enhancing plasticity, these DA changes are believed to modulate the learning process^33,34^. In line with this, the inhibition of cholinergic interneurons in the nucleus accumbens core (NAcc) augments cue-motivated behavior^24,35^. While the importance of ACh in learning processes is well recognized, the specific information encoded by ACh and its precise function remains unclear.

Recent evidence suggests that striatal cholinergic activity correlates with motivationally salient cues^36–38^. These cholinergic interneurons do not respond to neutral stimuli but develop a pause in their firing when stimuli become associated with rewards or punishments^36,38^. Based on these findings, we hypothesize that the ACh signal in NAcc represents cue salience. To investigate this hypothesis, we measured ACh and DA signals concurrently in the NAcc of rats via fiber photometry across four different odor discrimination experiments. We found that ACh developed characteristic dips to cues over learning before DA signals differentiated cues by value and modulated DA signal updates. In addition, these dips persisted even when cue value decreased and developed faster when rats solved similar problems in meta-learning tests. Computational modeling suggested that ACh signals reflect the salience changes predicted by a hybrid attentional associative learning model that integrated principles from the Mackintosh and Pearce-Hall models. Further training in a task with predictable odor sequences demonstrated that ACh signals encode cue salience rather than value expectation or motivation. Together, these results indicate that ACh in the NAcc encodes the salience of cues to regulate the learning rate.

## Results:

To investigate whether ACh in the NAcc core represents salience during associative learning, we recorded ACh signals using a genetically-encoded M3 receptor-based green fluorescent sensor, gACh4h^39^ (Figure S1a). Given the extensive studies linking NAcc DA to representing RPEs, we simultaneously recorded DA signals with a D1 receptor-based red fluorescent sensor, rDA3^40^ (Figure S1a) to identify the learning stages in conjunction with rat behaviors. These sensors were concurrently monitored in the NAcc of freely moving rats using fiber photometry (Figure S1b), allowing in vivo monitoring of neuromodulator signals with sub-second temporal resolution.

**Figure 1:**
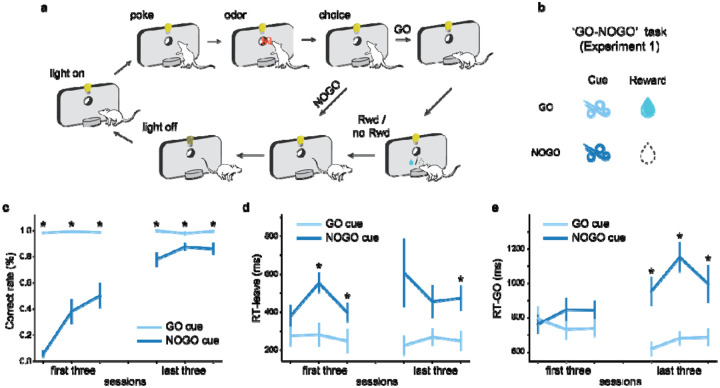
Go/No-Go task structure and behavioral performance. **a**. Schematic diagram of the basic structure during a trial across the backbone Go/No-Go contingency used in the four tasks applied in this study. **b**. Reward contingency for Experiment 1. Two cues are used in the Go/No-Go task. The GO cue indicates a water reward at the fluid well, while the NOGO cue indicates no reward. **c**. Percentage of correct trials (%). The correct rates of NOGO trials (dark blue line) increased across training sessions when and stayed high for the GO trials (light blue line). **d**. Latency to leave the central port. Over learning, after the termination of odor delivery, rats left the central port quicker following the GO cue compared to the NOGO cue. **e**. Latency to enter the fluid well. As learning progressed, rats became quicker at entering the fluid well after leaving the central port following the GO cue compared to the NOGO cue. The error bars in (**c-e**) indicate S.E.M. across rats. * indicates p < 0.05 in a two-tailed paired t-test. See also Figure S1.

The role of ACh in learning was tested in four distinct experiments, each using an odor-based go, no-go task design (Figure 1a). Trials began with the illumination of lights inside the training box. After a 500 ms delay following a nosepoke into the odor port, an odor cue was presented for another 500 ms. Rats could then leave the odor port and had 3 seconds to respond at the fluid well. If rats responded following rewarded cues, 0.05 ml of water was delivered after a 500 ms delay, and the lights turned off when the rat left the fluid well. If rats responded following non-rewarded cues, no fluid was delivered and the lights were turned off immediately, with an additional 4-second penalty added to the inter-trial interval (ITI). If no response was detected within 3 seconds, the lights turned off.

### ACh and DA show distinct dynamics during discriminative learning.

In Experiment 1, we explored the role of ACh during associative learning with a Go/No-Go task using the structure mentioned above with two distinct odors: a GO cue indicating a reward would be delivered and a NOGO cue indicating no reward (Figure 1b). Initially, rats responded at the fluid well after each cue presentation. Over time, they opted to withhold responses after the NOGO cue to avoid timeout penalties and maximize rewards over time (Fig, 1c). With 238.0 ± 63.6 trials in 2.86 ± 0.73 sessions on average, rats first reached 18 correct responses in a 20-trial moving block. The correct response was defined as responding at the fluid well after the GO cue but not the NOGO cue. Additionally, after learning rats exhibited longer reaction times when leaving the odor port and approaching to the fluid well following the NOGO cue over sessions, suggesting the NOGO cue elicited lower motivation (Figure 1d and 1e). These findings suggest that the rats successfully discriminated between the two olfactory cues and associated each cue with its corresponding reward outcome.

**Figure 2.**
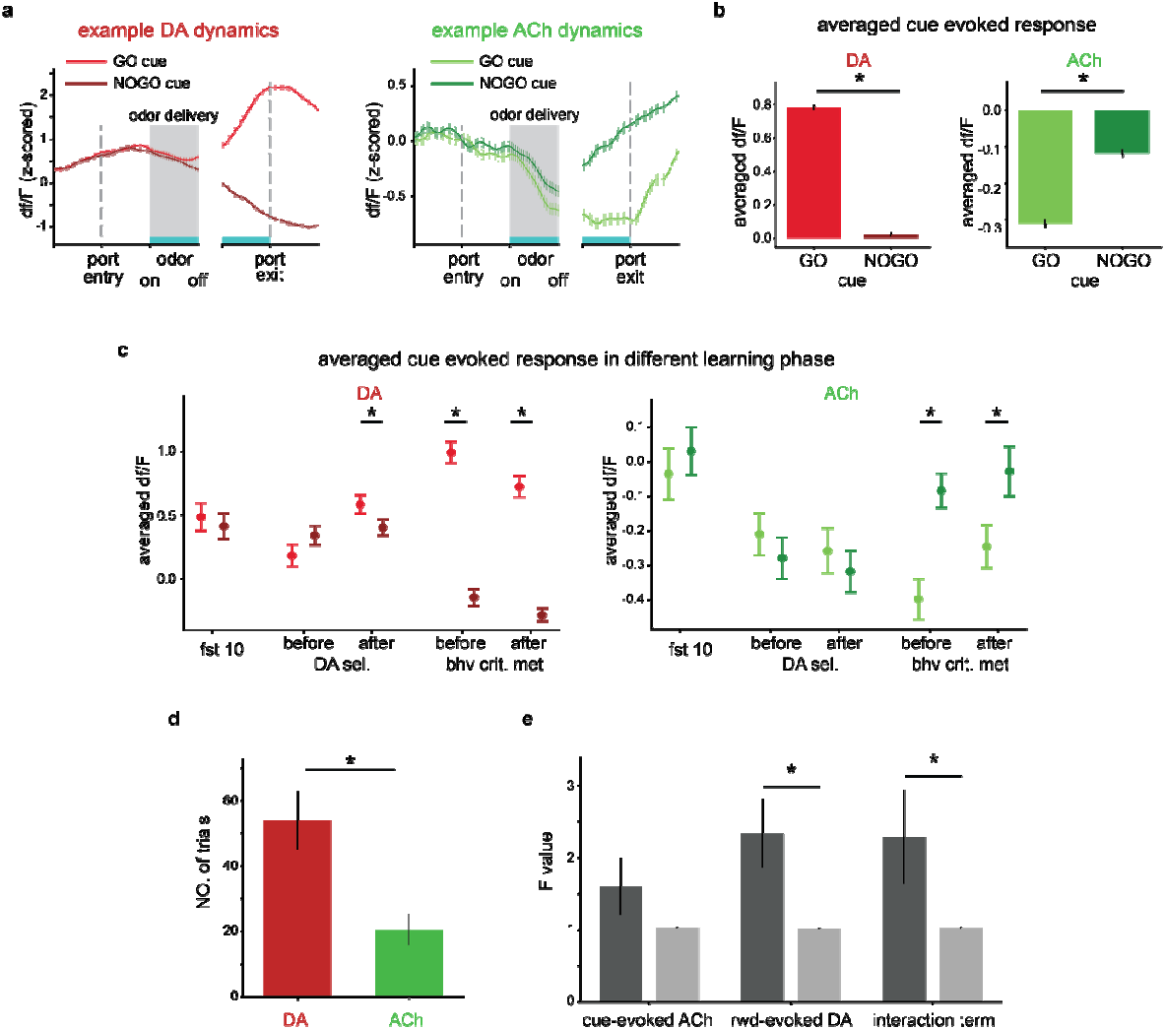
Dynamics of DA and ACh during the Go/No-Go task (^Experiment 1^). **a.** Example DA (left panel) and ACh (right panel) dynamics within trials. The light and dark lines represent signals in the GO and NO trials, respectively. DA responses increase over time in response to the GO cue and decrease during the NOGO cue trials, while ACh responses drop during the odor delivery for both cue types. The gray shaded areas indicate the odor delivery period. **b**. Averaged cue-evoked DA (left) and ACh (right) responses across all rats and sessions, computed during the period indicated by the blue bar on the x-axis in a. DA responses are significantly elevated during GO trials compared to the NOGO trials (=2.2e-278). ACh responses are significantly suppressed during both trial types and exhibit greater suppression during GO trials (=4.0e-30). **c**. Averaged cue-evoked DA (left) and ACh (right) signals across three learning milestones: learning initiation, the emergence of dopamine selectivity, and clear behavioral discrimination between cues. **d**. The number of trials required for the emergence of DA selectivity and ACh dips. The emergence of ACh dips precedes the DA selectivity (=0.013). **e**. F-values from the Granger causality test for the actual data (dark grey bars) versus shuffle data (light grey bars). Significant effects were found for reward-evoked DA responses (=1.9e-5) and its interaction with cue-evoked ACh responses (=0.0011) on the subsequent cue-evoked DA responses, but not for the cue-evoked ACh responses (=0.053). The error bars in (**b-e**) indicate S.E.M. across trials. * indicates p < 0.05 in a two-tailed t-test. Also see Figure S2 and S3.

We first examined the dynamics of ACh and DA signals by grouping trials based on presented odors. Aligning both signals from an example rat to the nose poke events that initiated new trials, we observed gradual increases in the DA signal before odor representation. The DA signal continued increasing after GO cues and decreased after NOGO cues (left panel, Figure 2a), consistent with its role in representing RPEs, while the ACh signals remained stable until rats entered the odor port and were then inhibited by both GO cue and NOGO cue (right panel, Figure 2a). Then, we quantified the cue-evoked response by averaging ACh or DA responses between odor delivery and odor port exit across all rats and sessions. As expected, DA responses were higher for the GO cue than the NOGO cue (left panel, Figure 2b, p=2.2e-278 in a two-tailed t-test). In contrast, ACh responses were significantly suppressed by both cues, with more pronounced dips for the GO cue (right panel, Figure 2b, p=4.0e-30).

To investigate whether cue-evoked ACh responses reflected changes in salience, we measured averaged ACh responses around three key learning milestones, defined by DA response and rat behavior. The first milestone occurred when rats were initially exposed to the olfactory cues. At this stage, the salience of the cues was minimal, as the rats had no prior knowledge of the association between cues and reward. The second milestone occurred when the DA signal first differentiated between two odor cues, indicating early learning that odors have different associations. At this stage, both GO and NOGO cues were expected to carry salience, as they were both predictive of outcomes: a time-out penalty following a response at the fluid well for NOGO cues, and a water reward for GO cues. In addition, no differentiation in their salience was expected since their different associations had only been recently learned. The third milestone demonstrated that rats showed a clear behavioral distinction between GO and NOGO cues, where rats should assign higher salience to the high-valued GO cue compared to the low-valued NOGO cue.

In the first stage, defined as the initial 10 trials when rats encountered the olfactory cues, DA responses were similar regardless of cue identity, consistent with the idea that the specific cue-outcome associations had not yet been established (left panel, Figure 2c. paired two-tailed t-test, p=0.49). During this period, ACh responses were statistically indistinguishable from zero for both GO and NOGO cues (right panel, Figure 2c. one sample t-test, p=0.66 and 0.63 for GO and NOGO trials, respectively). The second milestone was determined as when DA signals to GO and NOGO cues first became significantly different across 10 successive trials (paired two-tailed t-tests, p<0.01). We found that, before this point, ACh responses for both cues were significantly below zero (p=9.2e-6 and 8.1e-4 for GO and NOGO trials, respectively), with no significant difference between them (p=0.27). As expected, even when DA signals could discriminate between GO and NOGO, ACh responses still did not show cue selectivity (p=0.37). This suggests that DA responses differentiated cues earlier than ACh responses. The final milestone was quantified as when the correct rate first reached 90%. We found that ACh responses showed cue selectivity both in the 10 trials before (p=7.5e-6) and after (p=3.9e-4) this behavioral criterion was met. These findings were consistent with our hypothesis that ACh signal encoded the salience of cues.

If ACh indeed encodes salience, it should influence the updates of DA responses to the predictive cues, since salience modulates the learning rate by definition. This led to two key predictions. First, DA selectivity for cues should not emerge until cue-evoked ACh responses diverge from the baseline. To test this, we compared ACh responses against zero and DA responses to GO versus NOGO cues across every successive 10 trials for each rat. We found that cue-evoked Ach dips, defined as a significant drop below zero, emerged at approximately 20.54 ± 4.78 trials (mean values +/− S.E.M. across rats), whereas DA selectivity, defined as differential responses to GO versus NOGO cues, emerged significantly later at 53.92 ± 9.06 trials (Figure 2d, two-tailed t-test, p=0.013).

Second, ACh signal should influence the rate at which outcome-evoked prediction errors update the cue value. While optogenetic stimulation of NAc cholinergic interneuron during the cue representation period prevented cues from motivating action^24^, the quantitative relationship between ACh signaling and learning rate has not been characterized. Given the role of DA signals in representing RPE, reward-evoked DA responses could be used to estimate the reward-evoked prediction error, while changes in cue-evoked DA responses across trials could be used to estimate the updates in cue value. To test this prediction, we performed vector autoregression (VAR) analyses in which four sets of variables, i.e., cue-evoked ACh response, reward-evoked DA response, their interaction term, and the changes in cue-evoked DA from the previous $N_{\text{lag}}$ trials, were used to predict subsequent changes in cue-evoked DA signal for the same odor. The lag length, $N_{\text{lag}}=1$, was determined in advance using Bayesian information criteria (Figure S2a). Using Granger causality tests, we observed a significant effect of reward-evoked DA on subsequent cue-evoked DA responses compared to shuffled controls (two-tailed t-test, p=1.9e-5). Importantly, a strong and significant effect of the interaction term on subsequent cue-evoked DA responses was also detected (p=0.0011, Figure 2e), confirming that ACh responses modulated the learning rate and supporting the hypothesis that the ACh signal encodes the salience of cues.

### ACh responses in the Go/No-Go task were captured by a hybrid attentional associative learning model.

**Figure 3.**
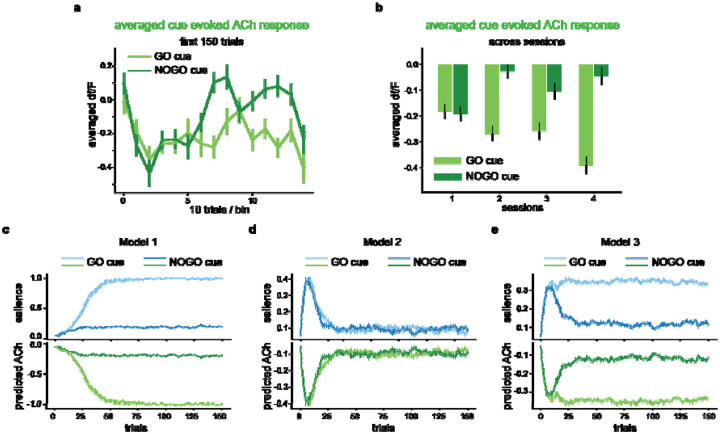
A hybrid attentional associative learning model captures biphasic dynamics and cue selectivity of the ACh responses. **a**. Average cue-evoked ACh responses for GO (light green) and NOGO (dark green) cues in the first 150 trials. **b**. Averaged cue-evoked ACh responses for GO (light green) and NOGO (dark green) cues across training sessions. The error bars indicate S.E.M. across trials. **(c-e)**. The upper panels show the dynamics of salience predicted by Model 1 **(c)**, based on the Mackintosh model; Model 2, based on the Pearce-Hall model **(d)**; and Model 3 **(e)**, which integrates salience mechanisms from both models. The lower panels show the ACh signal predicted by the models. The light lines indicate the GO cue, while the dark lines indicate the NOGO cue. The error bars indicate S.E.M. across runs. Also see Figure S4.

To further determine whether the ACh signal encoded salience, we analyzed the dynamics of ACh and compared it with salience according to different attentional associative learning models. We analyzed cue-evoked ACh changes over the first 150 trials for each condition and observed a biphasic trend: an initial gradual decrease followed by a subsequent rebound, especially for the response to the NOGO cue (Figure 3a). The subsequent rebound was unlikely caused by photobleaching, as the ACh response to the GO cue was mostly unchanged. To further rule out this possibility, we plotted the averaged ACh responses across sessions that should be affected by the photobleaching equally (Figure 3b). Results showed that the NOGO cue-evoked ACh changes in the first session were significantly more pronounced than in any subsequent session (p=2.0e-5, 0.038, and 6.2e-4 compared to the 2^nd^/3^rd^/4^th^ sessions, respectively). In contrast, the GO cue-evoked ACh changes in the first session were significantly less pronounced than in the second and fourth sessions but not significantly different from the third session (p=0.033, 0.083, and 4.3e-6 for comparisons with the 2^nd^/3^rd^/4^th^ sessions, respectively).

Our results have demonstrated that cue-evoked ACh responses modulate the updates of DA responses across trials, playing a role similar to that of salience. To investigate the nature of this contribution, we reconstructed the task using computational learning models derived from the attentional associative learning framework. In this framework, salience modulates the learning rate and, together with prediction errors, adjusts stimulus-outcome associations. However, the precise definition of salience varies across different models.

For example, Mackintosh’s widely used model assigns high salience to cues that are more predictive of outcomes than others, referred to as predictiveness-driven salience (PDS). The predictiveness-driven salience is further modulated by the absolute strength of the outcome, reflecting the fact that cues predicting higher reward value or stronger punishment retain greater salience. In contrast, the similarly influential Pearce-Hall model defines salience as the mean absolute value of the RPE over time, reflecting the uncertainty of the cue in predicting outcomes. This uncertainty-driven salience (UDS) reflects the fact that animals allocate more attention to and learn faster about cues with uncertain outcomes. We built models (see Methods for details of all models) grounded in these two models and performed 20 runs for each to ensure robustness. As ACh signals dipped in response to cues, we inverted the sign of the salience predicted by these models and compared it with the ACh response.

Model 1 grounded in the Mackintosh model, predicted an initial increase in salience for both GO and NOGO cues since they were the most reliable predictors among all perceivable stimuli (Figure 3c). The model also distinguished between the two cues by assigning higher boundary for the salience of the GO cue, which was associated with a more motivationally salient outcome, i.e., water reward, compared to the NOGO cue’s outcome, i.e., reward omission or timeout penalty. However, Model 1 exhibited a significant limitation: it predicted a monotonic increase in salience, which implied a corresponding monotonic decrease in the ACh responses (Figure 3c). This was inconsistent with the observed biphasic trend (Figure 3a), suggesting a critical shortcoming in Model 1 to capture the ACh dynamic.

Model 2, derived from the Pearce-Hall model, captured the dynamic of salience. Initially, the model did not learn the associations between presented cues and outcomes. Thus, a large RPE occurred upon outcome representation and enhanced cue salience. As learning progressed, the model learned the associations, eventually predicting reward outcomes accurately. As a result, salience for both GO and NOGO cues increased and subsequently decreased (Figure 3d). However, Model 2 predicted an identical salience pattern for GO and NOGO cues and failed to differentiate them (Figure 3a). Therefore, Model 2 was unable to capture the ACh selectivity during learning.

In Model 3, we mostly followed the framework proposed by Le Pelley et al, which integrates mechanisms from both the Mackintosh and Pearce-Hall models of salience. In this model, the salience was defined as the weighted sum of the PDS from the Mackintosh model and the UDS from the Pearce-Hall model. We found that the UDS component in Model 3 showed a biphasic pattern during learning, similar to Model 2. Specifically, the UDS initially increased with the prediction uncertainty when cue were newly introduced, then subsequently decreased as the cue-outcome associations were learned. Simultaneously, the PDS component increased and modulated by the learned value of cues, mirroring Model 1. As a result, the salience in the hybrid Model 3 matched both the biphasic dynamics and cue selectivity of ACh responses (Figure 3a and 3e).

To rule out the possibility that the distinct pattern of salience between models was from arbitrarily chosen parameters, we fitted models by minimizing the mean squared error in reproducing ACh responses with rat behavioral data. This optimization was performed using differential evolution. Due to the simulation’s complexity, we fitted learning rates for the association strength, PDS and UDS, which we believe most significantly affect the prediction error. A significantly smaller mean square error of reproducing the ACh responses by Model 3 was found compared to Model 1 (p=4.1e-59, two-tailed t-test) and Model 2 (p=4.4e-56). Thus, Model 3 comprehensively explained the observed ACh response patterns, indicating that ACh encodes the combined salience in both Mackintosh and Pearce-Hall models.

### ACh dynamics reflect cue-related salience changes after reward contingency reversal.

Experiment 1 and the simulations demonstrated that the ACh responses in a stationary environment followed the pattern predicted for salience. In Experiment 2a, we aimed to extend our findings and test if ACh tracks cue salience in a dynamic environment where reward contingencies change over time. In such a situation, the cue salience is found not to automatically update when the reward value changes^41,42^. For example, previous research has shown that the salience of a cue persists even after decreases in the value of the predicted outcome^43^. According to this, we hypothesized that after the reward contingency reversal in a Go/No-Go task, ACh dips to the previously rewarding cue should persist and dips to the previously non-rewarding cue should be enhanced due to its newly associated rewards, leading to comparable ACh responses to both cues. This pattern contrasts with the larger dips evoked by the rewarded cue observed before reversal.

**Figure 4.**
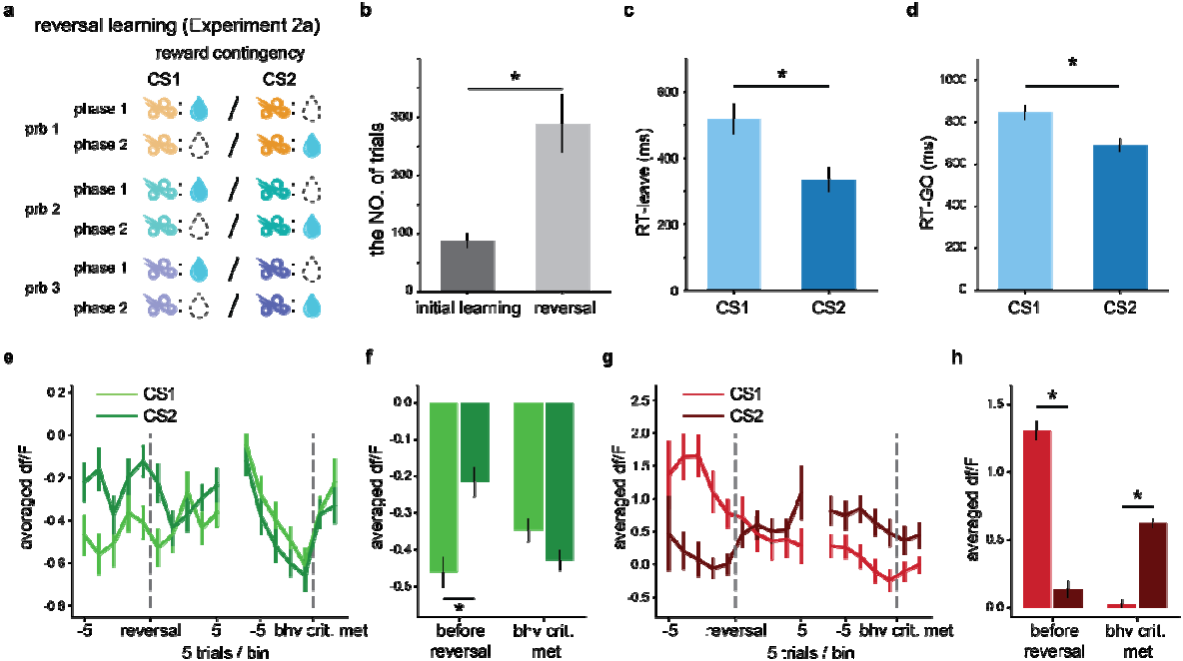
ACh responses to previously rewarded cue persisted after reversal (^Experiment 2a^). **a**. Schematic of reward contingencies in Experiment 2a. The reversal learning task is composed of three problems, each with a different odor pair. The reward contingencies are reversed during the learning. **b**. Number of trials required to reach the behavioral criterion during initial training (Phase 1) and after the reversal (Phase 2). **c**. Latency to leave the odor port following CS1 or CS2 presentation. **d**. Latency to enter the fluid well after CS1 or CS2. Rats exhibited shorter latencies for CS2 compared to CS1 after reversal. **e**. Dynamics of the ACh responses around the reversal point and upon reaching the reversal learning criterion are shown for CS1 and CS2. **f**. Average ACh responses to cues before the reversal and upon meeting the post-reversal behavioral criterion. **g**, **h**. DA signal dynamics and average responses. These panels follow the same format as panels (**e, f**), respectively. * in (**b-d, f, h**) indicates <0.05 in a two-tailed t-test.

To evaluate these predictions, we conducted Experiment 2a, which consisted of a series of three reversal problems (Figure 4a), each involving two distinct phases. In phase 1, conditional stimulus 1 (CS1) was rewarded, while conditional stimulus 2 (CS2) was non-rewarded. Training continued for an additional 20 trials after rats first achieved the behavioral criterion, which is 18 correct responses in a 20-trial moving block. Upon meeting the same criterion in the following session, we initiated phase 2, in which we reversed the reward contingencies associated with CS1 and CS2. Training with these reversed contingencies continued across multiple sessions until rats met the same behavioral criterion after reversal. After successfully completing the first reversal task, we repeated this two-phase process with second and third sets of odor pairs.

Rats reached the post-reversal behavioral criterion with training, but required more trials than the initial reward contingency learning (Figure 4b; 272.6 ± 44.8 vs. 95.3 ± 15.3 trials, respectively; paired two-tailed t-test, p=0.0011). After reversals, rats exhibited shorter response latencies in both leaving the odor port (Figure 4c, p=5.8e-4) and entering the fluid well (Figure 4d, p=5.1e-5) following CS2 compared to CS1. These behavioral changes suggested that after reversal, rats associated a higher value and greater motivation with CS2.

We averaged the signals around the reversal point and upon reaching the reversal learning criterion. The dynamics of the DA signal remained consistent with RPEs. As expected, before reversals, the cue-evoked DA response to CS1 was significantly larger than that to CS2 (p=1.9e-29, left side of Figure 4g and 4h). After the reversal, DA responses to CS2 rapidly increased and the responses to CS1 decreased (left side of Figure 4g). Eventually, the DA responses to CS2 became significantly higher than CS1 when the post-reversal behavioral criterion was met (right side of Figure 4h, p=3.3e-32).

The same procedure was applied to analyze ACh. Consistent with Experiment 1, before the reversal, both odors suppressed the ACh signal, with CS1 inducing a more pronounced dip compared to CS2 (left side of Figure 4e, p=3.4e-5). After the reversal, the magnitude of ACh dip evoked by CS2 increased while the dip evoked by CS1 persisted, resulting in statistically indistinguishable ACh responses to CS1 and CS2 (p=0.056). In contrast to the DA, even when rats reached the new criterion, the ACh responses still failed to differentiate between two cues (Figure 4f, p=0.062). These results were consistent with our predictions: the ACh responses to the previously rewarding cue (CS1) remained for an extended period and the responses to the newly rewarding cue (CS2) enhanced, leading to comparable ACh responses to CS1 and CS2 after the reversal.

**Figure 5.**
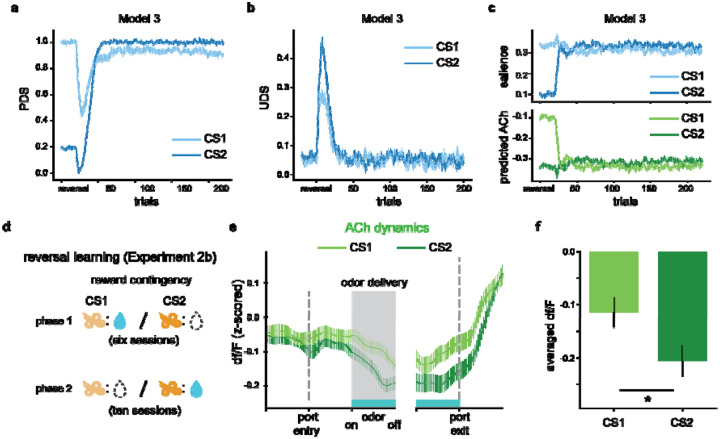
The hybrid associative learning model captured the dynamics of ACh signal in the reversal learning task (^Experiment 2b^). **(a-c)**. Dynamics of predictiveness-driven salience **(a)**, uncertainty-driven salience **(b)**, total salience (upper penal in **c)**, and ACh responses predicted (lower penal in **c)** by Model 3 following the reversal of reward contingencies. The light and dark lines represent signals in the CS1 and CS2 trials, respectively. Initially, reversal leads to elevated RPEs, leading to increases in PDS and decreases in UDS (blue arrows). With learning, the stimuli regain UDS due to improved relative predictiveness and lose PDS as uncertainty declines (purple arrows). The PDS and UDS changes of CS1 largely cancel out, while the new reward-associated CS2 shows a net increase in total salience due to enhanced UDS following the value update. The error bars indicate S.E.M. across runs. **d**. Schematic of reward contingencies in Experiment 2b. **e**. Empirical data showing the ACh dynamics within trials after extended post-reversal learning. **f**. Averaged cue-evoked ACh responses across all rats in the last three sessions, showing the reversed cue selectivity in ACh signaling. * indicates <0.05 in a two-tailed t-test.

Model 3 also captured the characteristics of ACh signal in Experiment 2a. After reversals, olfactory cues predicted the opposite reward outcome, leading to elevated RPEs and diminished predictiveness. Consequently, there was an increase in UDS and a decrease in PDS (blue arrows in Figure 5a and 5b). Once the values were updated, the stimuli regained PDS due to enhanced predictiveness and lost UDS owing to reduced uncertainty (purple arrows in Figure 5a and 5b). For CS1, the changes in PDS and UDS largely canceled each other, leading to little change in the overall salience (light lines in Figure 5c). However, CS2’s low pre-reversal UDS and its new association with water reward caused an increase in UDS, leading to a larger subsequent total salience (dark lines in Figure 5c).

The salience of CS1 remained high when the post-reversal behavioral criterion was met due to the slow decline in the upper boundary of UDS, reflecting the reward’s persistent influence. However, the model predicted that extended training would decrease this boundary, causing low UDS and reduced total salience for CS1. This prediction led us to hypothesize that extended post-reversal training would invert the preference of ACh responses in cues. To test this prediction, we conducted Experiment 2b (Figure 5d). In this experiment, five rats were trained in a Go/No-Go task for six sessions, and from the seventh session, we reversed the reward contingencies 20 trials after the rats achieved the behavioral criterion. Training with the reversed contingencies continued for ten sessions. Combining the data from the last three sessions, we observed a reversed selectivity in ACh responses: more pronounced dips in response to CS2, compared to CS1, were observed, as illustrated in Figure 5e and 5f (=0.025). These observations reveal the nature of ACh signaling in encoding cue salience and its adaptation to changing reward contingencies.

### Ach dynamics track accelerated salience acquisition in Meta-Learning

Meta-learning enables agents to generalize the knowledge acquired from previous problems to solve new ones efficiently. As in Experiment 1, rats learned that odors were predictive of rewards, a principle initially unknown to them. This acquired knowledge should allow rats to identify odor cues as salient more quickly when solving subsequent similar problems, thus accelerating the learning process. Consequently, we expected that the initial cue-evoked ACh responses, indicating the salience of cues, would show greater differences from baseline in later problems.

**Figure 6.**
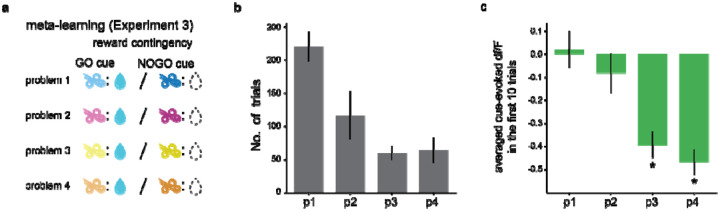
Generalization of task structure leads to earlier ACh cue responses (^Experiment 3^). **a**. Schematic reward contingencies in Experiment 3, consisting of four sequential Go/No-Go problems. Each problem introduces a unique pair of odors, one of which predicts a reward outcome. **b**. The number of trials needed to achieve criterion performance. It decreases across successive problems (Jonckheere–TeUDStra Test, p=1.3e–10), indicating that rats used previously acquired task structure to solve new problems. **c**. Average cue-evoked ACh responses in first 10 trials across problems. In the first and second problem, ACh responses are not significantly different from zero (One sample t-test, p=0.80 and 0.33 for 1^st^ and 2^nd^ problems, respectively), while in later problems, these responses become significantly negative (One sample t-test, p=9.8e-11 and 8.6e–15 for 3^rd^ and 4^th^ problems, respectively). * indicates p<0.05 in a one sample t-test.

To validate this prediction, we introduced Experiment 3, comprised of a series of four Go/No-Go problems (Figure 6a), each involving a unique pair of odors. Training for each problem was stopped 20 trials after rats achieved the behavioral criterion. Rats were then progressed to the next problem in the subsequent session until they had learned all four problems. Eight new rats were included in Experiment 3. To confirm that rats applied the previously acquired task structure to facilitate learning in new situations, we quantified the number of trials for rats to meet the criterion. A decreasing number of trials across successive problems was observed (Figure 6b, Jonckheere-TeUDStra Test, =1.3e-10), suggesting rats generalized the task structure to solve later problems.

The test our prediction regarding salience, we quantified the average cue-evoked ACh signals during the first ten trials. We found that in the first and second problems the ACh response did not significantly differ from zero (Figure 6c, one sample t-test, =0.80 and 0.33 for 1^st^ and 2^nd^ problems, respectively), mirroring observations in Experiment 1. However, in subsequent problems, the cue-evoked ACh response in these initial 10 trials became significantly lower than zero (One sample t-test, p=9.8e-11 and 8.6e-15 for the 3^rd^, and 4^th^ problems, respectively). Moreover, the ACh responses showed a monotonic divergence from the baseline across problems (Jonckheere-TeUDStra Test, p=4.4e-16). Collectively, these results showed that ACh dips occur increasingly earlier across problems during generalization, consistent with the prediction of salience.

### The ACh signal is independent of value expectation and motivation.

In both Go/No-Go and reversal tasks, after extensive training, the ACh signal became negatively correlated with DA signal, value expectation and motivation. While ACh dynamics significantly diverged from these variables during the learning phase, this dissociation could simply reflect a longer learning period required for ACh response development. Therefore, it is essential to determine whether, even after extensive training, the ACh response reflects cue salience rather than value expectation or motivation.

**Figure 7.**
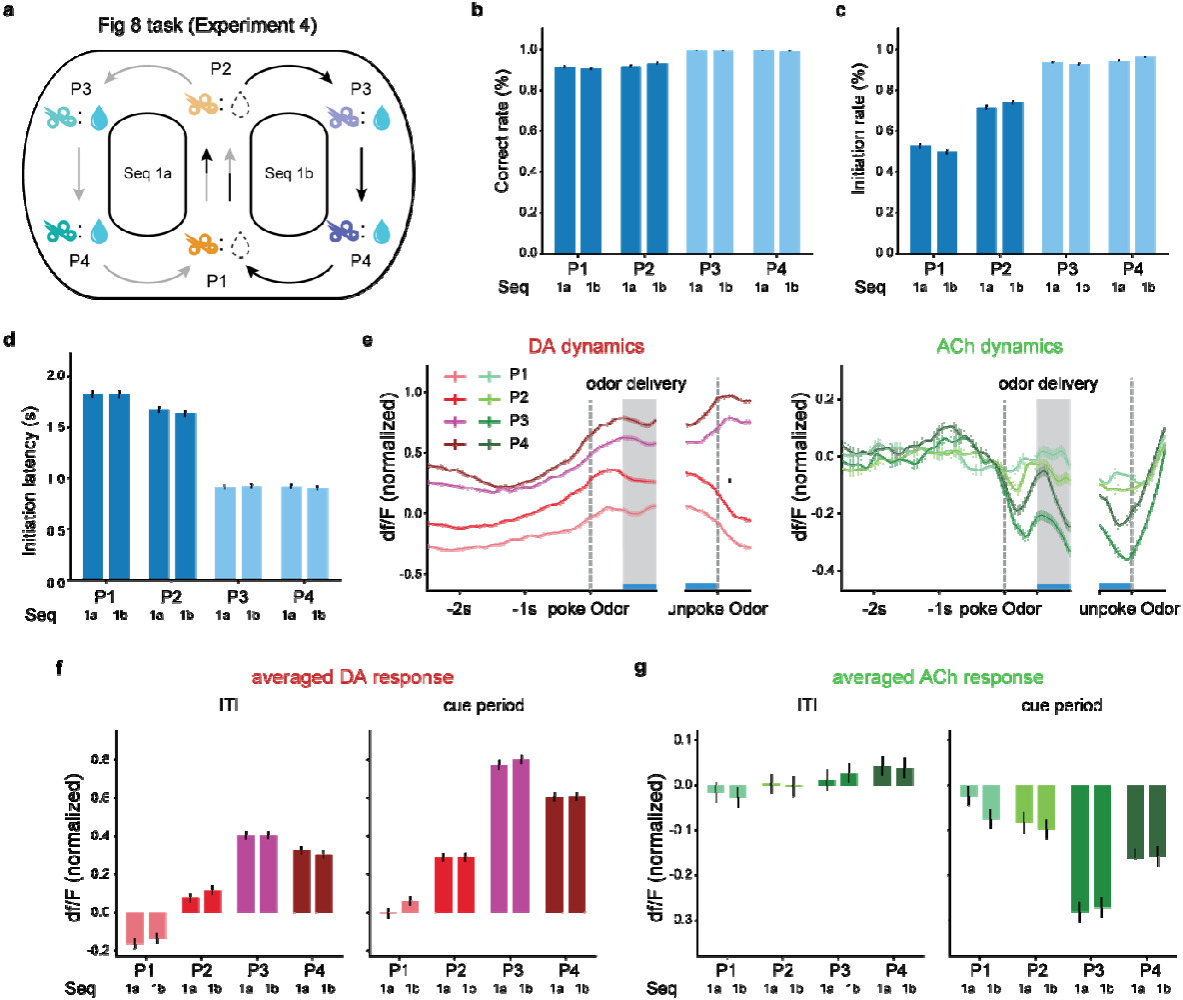
Ach signal is independent of value expectation and motivation (^Experiment 4^). **a**. Schematic odor sequences and reward contingencies in Experiment 4. This task consists of two distinct odor sequences (seq 1a and 1b), each with four positions (P1-P4). In the first two positions (P1, P2), identical and unrewarded odors are presented in both sequences. In the final two positions (P3, P4), sequence-specific and rewarded odors are presented. This design created predictable sequences with shared early cues but distinct reward-predictive outcomes. **b**. Percentage of correct trials (%) on each trial type in the sessions after rats first achieved an 80% correct rate in the NOGO trials. Dark blue indicates trial types without reward, while red indicates trial types with reward. **c**. Percentage of trials rats choosing to initiate a new trial after light onset. P1 vs P2 (=3.6e-57), P3 vs P4 (=7.1e-4) and <1e-100 for all other comparisons. **d**. Poke latency measures the time from light onset to odor port entry. Two-tailed t-tests: P1 vs P2 (=6.0e-6), P3 vs P4 (=0.81) and <1e-100 for all other comparisons. **e**. DA (left panel) and ACh (right panel) dynamics within trials. The gray shaded areas indicate the odor. **f**. The averaged DA response during the ITI cue period in each place. ITI: P1 vs P2 (=8.7e-23), P1 vs P3 (=8.9e-106), P1 vs P4 (p=4.9e-79), P2 vs P3 (p=4.0e-38), P2 vs P4 (p=4.9e-21), P3 vs P4(p=9.4e-5). Cue period: P1 vs P2 (p=7.8e-25), P1 vs P3 (p=8.1e-165), P1 vs P4 (p=3.4e-104), P2 vs P3 (p=3.5e-83), P2 vs P4 (p=9.6e-38), P3 vs P4 (p=1.3e-11). **g**. The averaged ACh response during the ITI and cue period in each place. ITI: P1 vs P2 (p=0.31), P1 vs P3 (p=0.067), P1 vs P4 (p=0.0060), P2 vs P3 (p=0.41), P2 vs P4 (p=0.078), P3 vs P4 (p=0.35). Cue period: P1 vs P2 (p=0.067), P1 vs P3 (p=1.9e-24), P1 vs P4(p=5.7e-7), P2 vs P3 (p=1.7e-16), P2 vs P4 (p=0.0019), P3 vs P4 (p=2.3e-07). The error bars in panels (**e-g**) indicate S.E.M. across trials. All p values are from two-tailed t-tests:

To address this concern, we used a ‘Figure eight’ task^44^ where odor cues were presented in a predictable, fixed sequence. Rats could predict future rewards across multiple trials before cue representation. This design dissociated value expectation and motivation from cue salience, as motivation and value expectation varied within the sequence before cue presentation when cue salience was zero. Therefore, if ACh signals encoded motivation or value expectation, they should differ before cue representation. Conversely, no differential response should be observed during this period, if ACh signals reflected cue salience,

In Experiment 4, one of six different odors was delivered in each trial. These six odors were arranged into two alternating sequences (seq 1a and seq 1b), each with four positions (P1–P4, Figure 7a). Two sequences shared identical non-rewarded odors in their first two positions (P1, P2) but contained sequence-specific rewarded odors in their last two positions (P3, P4). As expected, after learning, rats responded at the fluid well to GO cue presentation and withheld responses to the NOGO cue (Figure 7b). Moreover, rats initiated trials most frequently and rapidly when the reward was immediately available (P3, P4), moderately when the reward was one trial away (P2), and least when the reward was two trials away (P1). (Figure 7c and 7d) This systematic gradient in trial initiation rates and latencies suggested that rats’ subjective value expectation and motivation during ITI scaled with reward proximity within the sequences.

We first examined DA and ACh signals during the ITI (1s before the entry of the odor port) when rats already exhibited significantly different levels of value expectations and motivation. DA signals, known to reflect value expectation and motivation, were significantly modulated by the position: highest at reward-proximal positions (P3, P4), moderate at P2, and lowest at P1 (Figure 7f, left panel). In contrast, ACh signals were mostly constant regardless of positions (Figure 7g, left panel, only P1 vs P4 differed, p=0.0060). Only after odors associated with different saliences were delivered, ACh responses became position-dependent and showed an inverse relationship with cue salience and value expectation, which were correlated in this context, contrasting with the positive correlation of DA responses (Figure 7f and 7g, right panels). This pattern supported the salience hypothesis and argued against ACh encoding of value expectation or motivation. The disassociation between DA and ACh responses during the ITI also challenged previous proposals that ACh release was necessary to generate dopamine ramps^45^.

## Discussion:

We explored the role of ACh in the learning process by recording ACh and DA signals in the NAcc in multiple experiments. We found that ACh signals reflect a salience term that modulates learning rate, captured by a hybrid attentional associative learning model that integrates mechanisms from both the Mackintosh’s and Pearce-Hall models. Initially, the ACh signal did not change with presented novel cues, as these cues had not yet gained any salience. However, as cues acquired salience driven by predictiveness and uncertainty, characteristic ACh dips in response to cues emerged. The magnitude of these dips reflected the changing salience across different learning stages. In addition, the cue-evoked ACh dips persisted even when the value of cues decreased and appeared earlier when solving subsequent similar problems, consistent with theoretical predictions for salience. Further, we ruled out alternative hypotheses suggesting that ACh signals primarily encoded value expectation or motivation, as proposed by previous studies^25^.

While traditional reinforcement learning theories often assume a constant learning rate, effective learning requires adjusting this rate based on environmental volatility^46,47^. Recent studies suggest that dopamine transients encode RPEs independently of learning rates^48^, raising the question of how the learning rate is regulated. Previous studies suggested that the cholinergic system is also well-suited for this function. Indeed, extensive interactions between dopaminergic and cholinergic systems have been observed in multiple brain regions^22,23^. In addition, ACh dips in the NAcc have been proposed to create a permissive window for phasic dopamine increases and facilitate synaptic plasticity^33,49^, thus supporting the hypothesis that the ACh signal modulates the learning rate. Inspired by these findings, we directly demonstrated that ACh in NAcc followed the salience changes predicted by a hybrid attentional associative learning model and affected the rate of DA updates, which was aligned with the role of salience in regulating the learning rate. These results suggest a mechanism of how the learning rate is modulated in the brain and highlight ACh’s role in adaptive learning.

Although the accumbal cholinergic system has long been known to play a crucial role in learning, its precise computational role has remained uncertain. Our study suggests that ACh signals encode cue salience, which explains not only our recording results but also previous manipulation findings. For instance, inhibiting NAcc cholinergic interneurons enhances cue-motivated behavior, while stimulation diminishes it. These behavioral effects align with ACh’s proposed role in encoding salience and modulating learning rate: inhibiting cholinergic interneurons increases salience and learning rate, thereby augmenting cue-motivated behavior, while stimulation works the opposite.

This proposal contrasts with previous studies that have concluded that ACh signals in the dorsal striatum^50^ and basal forebrain^51^ encode RPEs. While the ACh signal in NAcc is also selective to cue value and decreases in response to reward outcomes as learning progresses, similar to an RPE signal, we believe that ACh in the NAcc does not directly reflect RPEs as in the basal forebrain. First, the ACh signal is unaffected by cues during the initial learning phase and becomes progressively inhibited by both GO and NOGO cues, carrying opposite RPEs. Second, DA signals, known to encode RPEs, develop cue selectivity earlier than the ACh signal does. Especially in the reversal learning task, the ACh signal could not differentiate cues even when rats learned the reversed reward contingency without further training. These observations suggest that ACh selectivity may be tied to more computationally complex variables like salience, rather than directly encoding RPE or value.

Importantly, the correspondence between ACh signals and the concept of cue salience does not rely on the specific form of the hybrid model used in this study. A similar model proposed by Esber and Haselgrove (2011)^6^, which also integrates PDS and UDS, produces similar predictions. Unlike the definitions in Mackintosh and Pearce-Hall models, PDS in this framework was defined as the sum of a cue’s association strength with positive and negative reinforcers, and UDS reflects the extent to which the cue itself is predicted by preceding events. Total salience is determined as the difference between these two components (Supp information). In Experiment 1, the PDS increased as cue-outcome associations were learned, and the UDS increased with repeated cue presentations. With an appropriate learning rate, total salience would initially increase and subsequently decline. Since the water reward supports stronger associations, the GO cue exhibited higher salience, consistent with predictions from our Model 3 (Figure S3). This robustness suggests that the salience carried by ACh signal reflects a more general principle of learning, transcending the particular model adopted here.

The associative learning model we used in the current study proposes that salience can modulate the learning rate and subsequent RPEs, while the RPEs quantify the prediction uncertainty and can, in turn, affect salience. Such a mechanism highlights potential interactions between cholinergic and dopaminergic systems across trials. Supporting this, we observed significant effects of cue-evoked ACh signal on the update of DA in the current study. However, we failed to observe a reciprocal effect of the DA signal on the ACh (Figure S2b and S3c), possibly due to the complexity of salience dynamics, which may obscure the effects of individual factors. The neural mechanisms underlying this modulation remain unclear and require further investigation.

In summary, our findings revealed that the ACh signal in NAcc encodes the salience of stimuli across various learning stages and tasks. We found surprising consistency between the ACh signal and salience predicted by a hybrid attentional associative learning model, where salience was determined by both predictiveness-driven and uncertainty-driven salience. These findings, together with the observed influence of ACh on DA updates, support ACh’s role in encoding salience and modulating learning rate throughout the learning process.

## RESOURCE AVAILABILITY

### Lead contact:

Further information and requests for resources and reagents should be directed to and will be fulfilled by the lead contact, Geoffrey Schoenbaum (geoffrey.schoenbaum@nih.gov).

### Materials availability:

- The data generated in this study will be deposited in the Zenodo database and accessible upon acceptance.
- All custom code used for reported analyses in this study will be available at GitHub upon acceptance.

## Methods:

### Experimental model and subject details

We included 23 male Long–Evans rats weighing over 450g at the start of the experiment, sourced from Charles River Laboratories and the NIDA IRP Breeding Facility. These rats were housed on a 12-h light-dark cycle at 25°C. They had free access to food but were water-restricted to approximately 85% of their original weight during the experiments. All rats received around 20 ml of water per day. Behavioral testing was performed during the light phase of the light-dark schedule. Experiment 1 was conducted on fourteen rats. One rat was excluded from the analyses due to markedly different ACh signals, although including this rat did not alter the overall conclusions. The ACh data for this rat, as well as the results for all fourteen rats combined, were provided in the supplementary materials (Figure S4). Of the fourteen rats, five were subsequently used for the long-term reversal learning study (Experiment 2b). An additional eight rats were initially used in Experiment 3, with five of them later also involved in Experiment 2a. One rat was not trained for Experiment 2a due to health issues, while two rats failed to meet the behavioral criterion in certain reversal problems and were therefore excluded from analyses. All procedures followed the National Institutes of Health guidelines, as determined by the National Institute on Drug Abuse Intramural Research Program (NIDA IRP) Animal Care and Use Committee (protocol no. 23-CNRB-108).

### Surgical procedures

Animals were anesthetized with isoflurane (3–5% for induction and 1–2% for maintenance). To simultaneously measure the dopamine and acetylcholine release in the nucleus accumbens core, we injected the D1 receptor-based red dopamine sensor-transfecting virus (pAAV-hsyn-rDA3m) and the muscarinic M3 receptor-based green acetylcholine sensor-transfecting virus (AAV-hSyn-gAch4h) into the unilateral NAcc of rats. The injection coordinates were AP +1.7 mm, ML ±1.7 mm, and DV −6.3 and −6.2 mm from the brain surface. Rats injected in the left and right brain were counterbalanced. In the five rats used for both Experiment 1 and the long-term reversal learning task, the DA and ACh sensor-transfecting viruses were injected into different hemispheres. For all other rats, both sensors were injected into the same hemisphere. A total of 1.0 μL of dopamine and acetylcholine sensor-transfecting virus, 0.5 μL for each, was delivered in each site at 0.1 μL/min via an infusion pump. All viruses were obtained from BrainVTA. The injection needle was held in for an additional 5 min after the completion of injection to avoid the backflow of viruses. An optic fiber (0.37 NA, 200μm diameter, Neurophotometrics, CA) was implanted in the most dorsal viral infusion location. Exposed fiber ferrules and a protective black 3D-printed headcap were secured to the skull with dental cement. After surgery, rats were given Cephalexin (15 mg/kg orally, once daily) for two weeks to prevent infections. Training started at least four weeks after the surgery for the virus expression.

### Fiber Photometry

#### Recording

Fluorescence signals were recorded using custom-ordered multi-pronged fiber optic patch cables (200 μm diameter, 0.37 NA, Doric Lenses, Canada) that were attached to the optic fiber ferrules on the skull of the rats with brass sleeves (Thorlabs, NJ). Up to 2 fibers were connected at a time in each rat for recordings, and they were shielded and secured with a custom 3D-printed headcap-swivel shielding system that allowed for the relatively free movement of the rats without the use of optic commutators and prevented the spillover of light during recordings.

Recordings were conducted using an FP3002 system (Neurophotometrics, CA), by providing fiber-coupled LEDs at 470nm (green, active acetylcholine-dependent signal), 560nm (red, active dopamine-dependent signal) and 415 nm (isosbestic reference signal) excitation light through the patch cord in interleaved LED pulses at 150 Hz (50 Hz acquisition rate for each channel). The light was reflected through a dichroic mirror and onto a 20×Olympus objective. Excitation power was measured at ~150–200 μW at the tip of the patch cord. Emitted fluorescent light was captured through the same cords, separated with an image splitting filter, and captured via a high quantum efficiency CMOS camera. Signals were acquired and synchronized with behavioral events using Bonsai.

#### Pre-processing

We filtered the raw fluorescence signals from the 470 nm (active), 560 nm (active), and 415 nm (reference) channels by using a causal median filter and a second-order Butterworth low-pass filter with a cutoff frequency of 5 Hz. Next, we fitted each channel’s data with a second-order Butterworth high-pass filter with a cutoff frequency of 0.002 Hz, aiming to eliminate the exponential decay in fluorescence caused by factors like photobleaching. After that, the reference channel data was then fitted to each active signal using second-order polynomial regressions, and the fitted data was subsequently subtracted from the active channel and divided by the exponential fit of the active channel to remove signal-independent variations in fluorescence. Finally, the resulting active signal was z-scored for each session.

### Behavioral task:

Rats were trained and recorded at least 4 weeks after the surgeries in standard operant boxes, which were aluminum chambers approximately 18 inches long in height, depth, and width. A central odor port was located above the two fluid wells located on the left wall of the chamber, and one well was physically blocked and not used during this study. Two lights were located above the odor port. The odor port was connected to an airflow dilution olfactometer to allow the rapid delivery and removal of olfactory cues. Odors were chosen from compounds obtained from International Flavors and Fragrances (New York, NY). Odor delivery and fluid delivery to the odor port and fluid well were controlled by the behavioral computer via a system of flow meters and solenoids. The entry into the odor port and fluid well was detected by the interruptions of an infrared beam, which was connected to the behavioral computer.

All experiments followed the same procedures in a single trial and differed only in the odor used and reward contingency. After the illumination of lights inside the box, rats were allowed to perform a nosepoke into the central odor port to initiate a new trial. Following a 500ms delay, an odor cue was presented for another 500ms. The presented odor was randomly selected from two possible cues that differed in different experiments and problems, and determined the reward availability in the fluid well. After the termination of odor delivery, the rats were free to leave the odor port and had 3 seconds to make a response at the fluid well. If a response was initiated, 0.05ml water was delivered 500ms later in reward trials, while no fluid was delivered in no-reward trials. During reward trials, the panel lights remained illuminated until the rat exited the fluid well, and the lights turned off indicated the end of a trial. In no-reward trials, the lights were turned off immediately after the response. If the rats did not respond within 3 seconds after leaving the odor port, the trial was classified as a no-go trial, and the lights were extinguished then. Inter-trial intervals commenced upon the turnoff of the panel lights and lasted for 4 seconds, extending to 8 seconds following responses in non-rewarded trials. Before involving any experiment, rats were trained with no odor and received water rewards at the fluid well to familiarize themselves with the task procedures.

#### Experiment 1:

Fourteen naive rats were included in experiment 1 and trained with a Go/No-Go task using the task structure mentioned above. Two distinct olfactory cues were used: one “GO” cue associated with a reward and the other “NOGO” cue associated with no reward (Figure 1b). To keep an overall balance in the number of positive vs negative trials throughout the session, we included 25 positive and 25 negative trials in every 50 trial block, but the order was pseudo-randomly generated independently. Rats were trained to learn the associations between cues and outcomes for at least six sessions.

#### Experiment 2a:

Seven naive rats were included in Experiment 3 and were then involved in Experiment 2a. Data from two were excluded from analyses since they failed to meet the behavioral criterion in certain reversal problems. Experiment 2a consisted of three reversal problems (Figure 5a), each involving two phases. In the first phase, the rats were trained with a Go/No-Go problem used in experiment 1. Once rats achieved the behavioral criterion on the initial problem, 18 correct responses within a 20-trial moving block, training was stopped 20 trials later to ensure that rats were equally proficient. The second phase was initiated once rats reached the criterion again in the next session. In the second phase, the reward contingencies between cues and outcomes are reversed, i.e., the cue previously associated with a reward now indicated no reward, and the cue previously associated with no reward led to a reward. To encourage rats to learn this reversal, we introduced three teaching trials after each reversal, in which the previously unrewarded cues were presented, and rats had unlimited time to respond at the fluid well. These teaching trials increased the probability that rats responded at the fluid well and discovered that the reward contingency was reversed. Training with reversed contingencies continued over multiple sessions until the rats met the same behavioral criterion. After the successful completion of the first reversal, the reward contingencies for the second and third sets of odor pairs were similarly trained and subsequently reversed.

#### Experiment 2b

Five rats used in Experiment 1 were trained with a Go/No-Go task over six sessions. In the seventh session, we reversed the reward contingencies 20 trials after the rats achieved the behavioral criterion. Training with the reversed contingencies then continued for ten sessions.

#### Experiment 3:

Eight naive rats were included in experiment 3. Each problem involved a unique pair of odors. Training for each problem was stopped after 20 trials whenever rats first achieved the behavioral criterion, a 90% success rate across 20 consecutive trials. Rats were then progressed to the next problem in the subsequent session until they had learned all four problems.

#### Experiment 4:

One naive rat and four rats were previously included in Experiment 3/2a and then involved in Experiment 4. On each trial, one of six different odors was delivered. These six odors were arranged into two alternating sequences (seq 1a and seq 1b), each with four positions (P1–P4). In the first two positions (P1, P2), identical and unrewarded odors were delivered in both sequences. In the final two positions (P3, P4), sequence-specific and rewarded odors were delivered. All rats were trained on the full set of sequences from Day 1.

### Histological procedures

After completion of the experiment, rats were perfused with chilled phosphate buffer saline (PBS) followed by 4% paraformaldehyde in PBS. The brains were then immersed in 20% sucrose in PBS for at least 24 hours and frozen. The brains were sliced at 50 μm in series for determination of optic fiber implant location in NAcc core and were then used for fluorescent immunohistochemistry. Brain slices were further stained with DAPI (Vectashield-DAPI, Vector Lab, Burlingame, CA), and processed for immunohistochemical detection of green and red fluorescent protein. For fluorescence immunohistochemistry, gACh4h was immunostained using a chicken anti-GFP antibody (1:1,000, Abcam, catalog #ab13970) in 0.1% Triton X-100/1× PBS and incubated in Alexa Fluor 488 AffiniPure Donkey Anti-Chicken IgY (IgG) secondary antibody (H+L, code: 703-545-155, RRID: AB_2340375, dilution: 1:100) overnight. rDA3m was immunostained using a rabbit anti-RFP antibody (1:1,000, Rockland, catalog #600-401-379) in 0.1% Triton X-100/1× PBS and followed by incubated in Alexa Fluor 594 AffiniPure Donkey Anti-Rabbit IgG secondary antibody (H+L, code: 711-585-152, RRID: AB_2340621, dilution 1:100). Fluorescent microscopy images of the slides were acquired with a BZ-X800 Keyence microscope. Expression patterns were extracted from the images and then superimposed on anatomical templates.

### Vector autoregression (VAR) modeling and Granger causality testing

The hypothesis that the level of ACh release evoked by a cue modulates the learning rat led to the prediction that the ACh release, in combination with the prediction error induced by the reward outcome, could significantly influence the updating of the cue’s value. Prediction error evoked by the reward outcome can be estimated by the corresponding DA response, while the cue’s value updates can be inferred from changes in the cue-evoked DA response across trials.


$$DA_{\text{cue}}^{t+1}-DA_{\text{cue}}^{t}\sim \left(ACh_{t}^{\text{cue}}+{\text{baseine}}_{1}\right)*\left(DA_{t}^{\text{reward}}+{\text{baseline}}_{2}\right)$$

To test this relationship, we used the first 150 trials for each condition and performed a Vector Autoregression (VAR) model to analyze the correlation between the cue-evoked DA response, the cue-evoked ACh response, the reward-evoked DA response, and their interaction term. The model is expressed as:

$$DA_{\text{cue}}^{t+1}-DA_{\text{cue}}^{t}=c+\sum_{n=0}^{\text{lag}}k_{1}*\left(DA_{\text{cue}}^{t-n}-DA_{\text{cue}}^{t-n-1}\right)+k_{2}*ACh_{\text{cue}}^{t-n}+k_{3}*DA_{\text{reward}}^{t-n}+k_{4}*DA_{\text{reward}}^{t-n}*ACh_{\text{cue}}^{t-n}+\varepsilon ,$$

where c is a constant, $k_{i}$ are model parameters and ε is the error term. Bayesian information criterion (BIC) was calculated for different lags, and the lag with the lowest information criterion value was selected for further analysis (Figure S2a).

Next, the model was fitted separately for each session and trial type (presented odor). Granger causality analysis was then applied to determine whether the cue-evoked ACh response, reward-evoked DA response, and their interaction term provided significant information about the cue-evoked DA response beyond what could be explained by its past values. The F values were from the original data compared to those obtained from the control data, where the $DA_{\text{cue}}$ were shuffled 1000 times, to determine the statistical significance of each factor’s effect on cue-evoked DA responses.

### Attentional associative learning models

We used three attentional associative learning models to simulate the salience dynamics of stimuli in the learning process. The mechanisms for updating associative strength were the same across models, differing only in how salience was defined. By varying the definition of salience, three models provide different predictions on the salience dynamics during learning.

The associative strength for a conditioned stimulus, A, was represented by an excitatory component, $V_{A}$, and an inhibitory component, ${\overset{‾}{V}}_{A}$, indicating the expectation of positive (water reward) and negative reinforcer (timeout), respectively. The net associative strength, $V_{A}^{\text{net}}$, is calculated as:

$$V_{A}^{\text{net}}=V_{A}-{\overset{‾}{V}}_{A}.$$

The updates to these components are driven by the prediction error, $\delta_{v}$, defined as:

$$\delta_{v}=\lambda -\sum_{X}V_{X}^{\text{net}},$$

where X represents the set of stimuli represented in the environment and λ is the signed maximum associative strength supported by the reinforcer, representing either the reward or penalty value. Specifically, λ is set to 1 for water reward, −0.1 for reward omission, and −0.2 for timeout penalty. The models update the excitatory and inhibitory components of the associative strength based on the sign and magnitude of the $\delta_{v}$,

$$\left\{\begin{array}{cc} V_{A}=V_{A}+\eta_{V}\cdot S_{A}\cdot \delta_{v}+N\left(0,0.025\right), & \text{if}\;\delta_{v}>0\\ {\bar{V}}_{A}={\bar{V}}_{A}+\eta_{V}\cdot S_{A}\cdot \delta_{v}+N\left(0,0.0.25\right), & \text{if}\;\delta_{v}>0 \end{array}\right.$$

where $\eta_{V}$ is the learning rates for associative strength, $S_{A}$, indicating the salience of stimulus A, modulates the overall impact of the $\delta_{v}$ on the associative strength and N(0,0.025) is the Gaussian noise with mean 0 and variance 0.025.

#### Model 1:

Model 1 is grounded in the Mackintosh model. The salience, S, is determined by the predictiveness, α, of a stimulus. Stimuli providing higher predictiveness compared to other perceivable stimuli are assigned higher salience. The change in $\alpha ,\delta_{\alpha }$, is defined as the difference between the absolute error for other stimuli (X≠A) and the absolute error for the target stimulus (A):

$$\delta_{\alpha }=\left|\lambda -\sum_{X\neq A}V_{X}^{\text{net}}\right|-\left|\lambda -V_{A}^{\text{net}}\right|$$

$$\alpha =\left\{\begin{array}{ll} \alpha +\eta_{\alpha }^{\text{pos}}\cdot \delta_{\alpha }+N(0,0.05), & \text{if}\delta_{\alpha }>0\\ \alpha +\eta_{\alpha }^{\text{neg}}\cdot \delta_{\alpha }+N(0,0.05), & \text{if}\delta_{\alpha }<0 \end{array}\right.$$

where $\eta_{\alpha }^{\text{pos}}$ and $\eta_{\alpha }^{\text{neg}}$ are the learning rates for increasing and decreasing α, respectively. To ensure that α remains within a meaningful range, it is rectified according to an upper limit, $\alpha_{\text{upper}}$, and a lower limit, $\alpha_{\text{lower}}$. If α exceeds the upper limit, it is set to the upper limit, and if it drops below the lower limit, it is set to the lower limit. $\alpha_{\text{lower}}$ is pre-defined, while $\alpha_{\text{upper}}$ is dynamic and determined by the absolute strength of the reinforcer.


$$\alpha_{\text{upper}}=\left\{\begin{array}{cc} 0.9995\cdot \alpha_{\text{upper}}, & \text{if}|\lambda |<\alpha_{\text{upper}}\\ |\lambda |, & \text{if}|\lambda |>\alpha_{\text{upper}} \end{array}\right.$$

#### Model 2:

Model 2 is grounded in the Pearce-Hall model. The salience of a stimulus is determined by its uncertainty. Stimuli, which provide lower predictiveness and indicate higher uncertainty about the upcoming observations, are assigned higher salience, σ. The error in $\sigma ,\delta_{\sigma }$, is defined as:

$$\delta_{\sigma }=\left|\lambda -\sum_{X}V_{X}^{\text{net}}\right|$$

Then σ is then updated similarly to α, and the learning rate varies based on whether $\delta_{\sigma }$ is positive or negative:

$$\sigma =\left\{\begin{array}{ll} \left(1-\eta_{\sigma }^{\text{pos}}\right)\cdot \sigma +\eta_{\sigma }^{\text{pos}}\cdot \delta_{\sigma }+N(0,0.05), & \text{if}\delta_{\sigma }>0\\ \left(1-\eta_{\sigma }^{\text{neg}}\right)\cdot \sigma +\eta_{\sigma }^{\text{neg}}\cdot \delta_{\sigma }+N(0,0.05), & \text{if}\delta_{\sigma }<0 \end{array}\right.$$

#### Model 3:

Model 3 integrates salience components from both the Mackintosh and Pearce-Hall models, The salience, S, is determined by the weighted sum of predictiveness-driven and uncertainty-driven salience of a stimulus.

S=ω⋅α+(1-ω)⋅σ

where 0<ω<1 determines the relative contribution of α and σ. In all simulations, ω is set to 0.3. The dynamics and update rules of α and σ are the same as those described in the Model 1 and Model 2.

#### Go/No-Go task

We simplified the task structure in our simulations. Completed trials from rats were used to train the models. For each trial, we assumed that two stimuli were represented: the presented odor and the background stimuli in the environment. Following these cues, one of three reinforcers was presented: water reward, reward omission, and timeout penalty. In all three models, $\eta_{V}=0.8,\eta_{\alpha }^{\text{pos}}=\eta_{\alpha }^{\text{neg}}=0.3$, and $\eta_{\sigma }^{\text{pos}}=\eta_{\sigma }^{\text{neg}}=0.5$.

To evaluate the goodness of fit for ACh signal in the Go/No-Go task, we used rat behavioral data and fitted models by minimizing the mean squared error in reproducing ACh responses. This optimization was performed using differential evolution. Due to the simulation’s complexity, we fitted three key free parameters in each model that we believe most significantly affected the results. For Model 1, we relaxed the assumption that $\eta_{\alpha }^{\text{pos}}=\eta_{\alpha }^{\text{neg}}$ and set $\eta_{\sigma }^{\text{pos}}=\eta_{\sigma }^{\text{neg}}=0$. The fitted parameters were $\eta_{V},\eta_{\alpha }^{\text{pos}}$ and $\eta_{\alpha }^{\text{neg}}$. For Model 2, we relaxed the assumption that $\eta_{\sigma }^{\text{pos}}=\eta_{\sigma }^{\text{neg}}$, and set $\eta_{\alpha }^{\text{pos}}=\eta_{\alpha }^{\text{neg}}=0$. The fitted parameters were $\eta_{V},\eta_{\sigma }^{\text{pos}}$ and $\eta_{\sigma }^{\text{neg}}$. For Model 3, we assumed $\eta_{\alpha }^{\text{pos}}=\eta_{\alpha }^{\text{neg}}=\eta_{\alpha }$ and $\eta_{\sigma }^{\text{pos}}=\eta_{\sigma }^{\text{neg}}=\eta_{\sigma }$. The fitted parameters were $\eta_{V},\eta_{\alpha }$ and $\eta_{\sigma }$. We compared the losses from all entries in the population generated by the fitting algorithm, i.e., differential evolution, across different models to evaluate their relative fitting performance.

#### Reversal task

To capture the dynamics of the ACh signal in the reversal task, we relaxed the assumptions $\eta_{\alpha }^{\text{pos}}=\eta_{\alpha }^{\text{neg}}$ and $\eta_{\sigma }^{\text{pos}}=\eta_{\sigma }^{\text{neg}}$. The results shown in Figure 5 were obtained using the parameters: $\eta_{\alpha }^{\text{pos}}=0.3$ and $\eta_{\sigma }^{\text{pos}}=0.15$. All other parameters were consistent with those used in the Go/No-Go task.

## Supplementary Material

Supplement 1
