# Accumbal acetylcholine signals associative salience during learning

**DOI:** 10.1101/2025.01.06.631529

**Published:** 2025-04-25

**Authors:** Zhewei Zhang, Kauê Machado Costa, Yizhou Zhuo, Guochuan Li, Yulong Li, Geoffrey Schoenbaum

**Affiliations:** 1 National Institute on Drug Abuse Intramural Research Program, National Institutes of Health, Baltimore, MD, 21224, USA.; 2 Department of Psychology, University of Alabama Birmingham, Birmingham, AL, 35223, USA.; 3 State Key Laboratory of Membrane Biology, School of Life Sciences, Peking University, Beijing 100871, China.; 4 PKU-IDG/McGovern Institute for Brain Research, Beijing, 100871, China.; 5 Peking-Tsinghua Center for Life Sciences, New Cornerstone Science Laboratory, Academy for Advanced Interdisciplinary Studies, Peking University, Beijing, 100871, China.; 6 Chinese Institute for Brain Research, Beijing, 102206, China.; 7 Institute of Molecular Physiology, Shenzhen Bay Laboratory, Shenzhen, Guangdong, 518055, China.; 8 National Biomedical Imaging Center, Peking University, Beijing, 100871, China.

## Abstract

Learning is driven by prediction errors, which determine what is learned, and salience, which controls the learning rate. Dopamine in the nucleus accumbens correlates with prediction errors, but salience mechanisms are less clear. We hypothesized that acetylcholine acts as a salience signal, as it regulates dopamine-induced plasticity. To test this, we recorded acetylcholine and dopamine dynamics in rats across three learning tasks. Our findings reveal a characteristic pattern of evolving neuromodulator responses during learning. First, indiscriminate cholinergic dips appeared, then dopamine responses differentiated based on predicted value, followed by the cholinergic dips also varying by value. Stable performance required this full pattern, and acetylcholine dips could be decoupled from value by changing reward contingencies. The observed acetylcholine dynamics fit an associative salience term predicted by hybrid attentional associative learning models, suggesting that dopamine and acetylcholine act complementarily to determine the content and rate of learning.

## Teaser

Acetylcholine shapes learning rate by signaling the salience of predictive cues.

## Introduction

Learning is critical for survival in natural environments, where determining which cues can reliably predict relevant outcomes, like the presence of food or the approach of predators, is paramount. Learning of the associations between cues and outcomes is driven by prediction errors, the difference between known expected and actual outcomes. Animals also learn to attribute salience to cues [1–3], and cues support faster learning. These two processes are intrinsically intertwined: learning about the outcome that follows a cue influences its salience, and the salience of a cue, in turn, affects the rate of learning subsequent associations to that cue.

These learning processes have been formalized in attentional associative learning models [4–7]. Two influential models, the Mackintosh[3, 4] and Pearce-Hall models[7, 8], emphasized different aspects of salience dynamics. Mackintosh proposed that a stimulus gains salience if it reliably predicts an outcome[9, 10]. This model explains various phenomena, such as the observation that previously blocked cues or dimensions are slower to support novel associations[11, 12]. In contrast, the Pearce-Hall model posits that salience is assigned to cues paired with uncertain outcomes, as a mechanism to minimize uncertainty about the environment[13, 14]. This model accounts for phenomena like latent inhibition[15, 16], where repeated exposure to an isolated cue impairs subsequent learning about associations to that cue. However, accumulating evidence suggests that salience is affected by both predictiveness and uncertainty, which has led to the proposal of hybrid models[6, 17] that integrate salience components from both Mackintosh and Pearce-Hall models. Le Pelley (2004)[17] and Esber and Haselgrove (2011)[6] defined and integrated these two components to determine a cue’s ultimate total salience, explaining a broader range of experimental findings.

Regarding the neurobiological mechanisms of learning, striatal dopamine (DA), especially in the nucleus accumbens core (NAcc), has long been regarded as a neural representation of the prediction error signals thought to underlie learning [18–20]. This is especially true for reward prediction errors (RPEs), which are the differences between predicted and experienced reward, or value. Current theory posits that DA drives learning by modulating synaptic plasticity in target circuits in proportion to the magnitude of experienced RPEs. However, it remains unclear whether associative salience might also be conveyed by a similar neuromodulatory mechanism. One candidate neuromodulator for this function is acetylcholine (ACh). Most striatal cholinergic neurons are tonically active but pause their firing in response to rewards and reward-predictive cues[21, 22], and we recently demonstrated that ACh release in the NAcc also dips in response to these events[23]. Similar patterns have been observed in other regions of the striatum[24, 25], and inhibition of cholinergic interneurons in the NAcc also augments cue-motivated behavior[26, 27]. While there is some conflicting evidence[28, 29] on this matter, the majority of the literature indicates that ACh typically dips during the periods where DA would signal RPEs[25, 30, 31]. Moreover, at the cellular level, ACh typically acts in opposition to DA in its effects on synaptic plasticity, and is therefore well positioned to gate or modulate DA-induced plasticity[32, 33], as would be expected of a salience signal. However, the specific information encoded by ACh and its precise algorithmic function during learning remain unclear.

Here, we tested the hypothesis that accumbal ACh conveys an associative salience signal. We did this by simultaneously recording ACh and DA signals in the NAcc of rats across three different odor discrimination learning tasks. We found that the characteristic ACh dips in response to cues develop over learning. These dips typically appeared before the development of differential DA responses to rewarded and unrewarded cues (a hallmark of RPE encoding), and were initially of the same magnitude for rewarded and unrewarded cues. Over time, DA responses to rewarded and unrewarded cues diverged, and after that the magnitude of the Ach dips to the non-rewarded cue decreased. These changes in DA and ACh signals occurred before the rats reached proficiency in the task, suggesting that this dynamic neuromodulatory progression underlies learning. In line with this interpretation, further analyses indicated that ACh dips directly promoted changes in DA signals and behavioral learning progression. Moreover, these dips persisted even when cue values were decreased, tracking the history of previous associations, which demonstrated that ACh dips did not represent only value or RPEs. Instead, computational modeling demonstrated that ACh dips reflect associative salience, as predicted by a hybrid attentional associative learning model that integrated principles from the Mackintosh and Pearce-Hall models. Together, these results indicate that ACh in the NAcc encodes the learned salience of cues to regulate learning rate.

## Results

To investigate whether ACh in the NAcc core represents salience during associative learning, we recorded ACh signals using a genetically-encoded green fluorescent sensor, gACh4h[23] (fig. S1A). Given the link between NAcc DA and RPE representation, we simultaneously recorded DA signals with a red fluorescent sensor, rDA3[34] (fig. S1A), as a concurrent measure of behavioral teaching signals. These sensors were monitored in the NAcc of freely-moving rats using fiber photometry (fig. S1B), allowing in vivo monitoring of neuromodulator signals with sub-second temporal resolution.

**Fig. 1.**
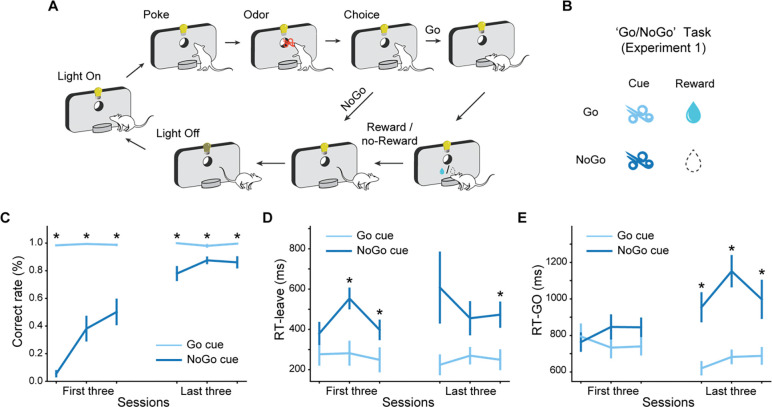
Go/NoGo task structure and behavioral performance. (**A**) Schematic diagram of the basic structure during a trial across the backbone Go/NoGo contingency used in the four tasks applied in this study. (**B**) Reward contingency for Experiment 1. Two cues are used in the Go/NoGo task. The Go cue indicates a water reward at the fluid well, while the NoGo cue indicates no reward. (**C**) Percentage of correct trials (%). The correct rates of NoGo trials (dark blue line) increased across training sessions when and stayed high for the Go trials (light blue line). (**D**) Latency to leave the central port. Over learning, after the termination of odor delivery, rats left the central port quicker following the Go cue compared to the NoGo cue. (**E**) Latency to enter the fluid well. As learning progressed, rats became quicker at entering the fluid well after leaving the central port following the Go cue compared to the NoGo cue in the few trials where rats responded to the NoGo cue. The error bars in (C-E) indicate S.E.M. across rats. * indicates p<0.05 in a two-tailed paired t-test.

The role of ACh in learning was tested in three distinct experiments, each using an odor-based Go, NoGo task design (fig. 1A). In each trial, rats sampled an odor presented at a centrally located port and then decided whether to respond at a nearby fluid well based on the presented odor. Responses to rewarded cues resulted in a water reward, while responses to non-rewarded cues resulted in no reward and timeout penalties.

### ACh and DA show distinct dynamics during discriminative learning.

In Experiment 1, we explored the role of ACh during associative learning with a Go/NoGo task using the structure mentioned above with two distinct odors: a Go cue indicating a reward would be delivered and a NoGo cue indicating no reward (fig. 1B). Initially, rats responded at the fluid well after each cue presentation. Over time, they learned to withhold responses after the NoGo cue to avoid timeout penalties and maximize reward acquisition over time (fig, 1C). Rats were considered proficient when they reached 18 correct responses in a 20-trial moving block, which they accomplished on average within 238.0 ± 63.6 trials in 2.86 ± 0.73 sessions. Correct responses were defined as responding at the fluid well after the Go cue and not entering the fluid well after the NoGo cue. In addition to increasing accuracy, over the course of learning, the rats progressively decreased their response reaction times following the Go cue and increased it after the NoGo cue, both for leaving the odor port and entering the fluid well (fig. 1D and E). These findings show that the rats successfully discriminated between the two odor cues and associated each with the corresponding reward outcome.

**Fig. 2.**
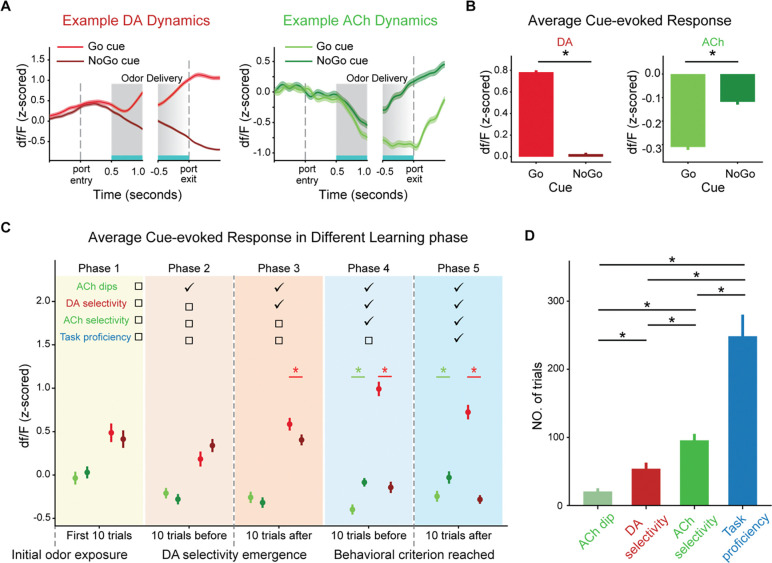
Dynamics of DA and ACh during the Go/NoGo task (Experiment 1). (**A**) Example DA (left panel) and ACh (right panel) dynamics within trials. The light and dark lines represent signals in the Go and NO trials, respectively. DA responses increase over time in response to the Go cue and decrease during the NoGo cue trials, while ACh responses drop during the odor delivery for both cue types. The gray shaded areas indicate the odor delivery period. The solid gray box marks the first 500 ms after odor onset, where odor delivery is present in all trials. The gradient gray box represents the last 500 ms before port exit, where odor presence varies. In trials where rats left the port quickly after odor offset, odor was still present, whereas in slower trials, it had already ceased. (**B**) Averaged cue-evoked DA (left) and ACh (right) responses across all rats and sessions, computed during the period indicated by the blue bar on the *x*-axis in (A). DA responses are significantly elevated during Go trials compared to the NoGo trials (p=2.2e-278). ACh responses are significantly suppressed during both trial types and exhibit greater suppression during Go trials (p=4.0e-30). (**C**) Averaged cue-evoked DA (left) and ACh (right) signals across three learning milestones: learning initiation, the emergence of dopamine selectivity, and clear behavioral discrimination between cues. The error bars in (B and C) indicate S.E.M. across trials. (**D**) The number of trials required for the emergence of ACh dips, DA selectivity, ACh selectivity and reaching the behavioral criterion. The emergence of ACh dips occurs first, followed by DA selectivity, then ACh selectivity, and finally, the achievement of the behavioral criterion. The error bars in (D) indicate S.E.M. across rat. Each * indicates p<0.05 in a two-tailed t-test.

We first examined the dynamics of ACh and DA signals by grouping trials based on presented odors. DA signals gradually increased before odor onset and continued to rise after the representation of the Go cue, while they decreased following the NoGo cue (left panel, fig. 2A), consistent with its role in representing RPEs. In contrast, the ACh signals did not deviate from baseline until rats entered the odor port, as we previously reported[23], and were then inhibited by both Go cue and NoGo cue (right panel, fig. 2A). We quantified the cue-evoked response by averaging ACh or DA signals between odor delivery and odor port exit, including the period after odor offset when the rat remained in the port, across all rats and sessions. As expected, DA responses were higher for the Go cue than the NoGo cue (left panel, fig. 2B, p=2.2e-278 in a two-tailed t-test). In contrast, ACh responses were significantly suppressed by both cues, with more pronounced dips for the Go cue (right panel, fig. 2B, p=4.0e-30).

To investigate whether the dynamics of cue-evoked ACh responses tracked changes in salience over learning, we measured ACh responses in five phases defined by three key behavioral and neural milestones. The first milestone occurred when rats were initially exposed to the odor cues. At this point, the salience of the cues was minimal, as the rats had no prior knowledge of the association between cues and reward. The second milestone was determined as when DA responses to Go and NoGo cues first became significantly different across 10 successive trials (paired two-tailed t-tests, p<0.01), which was a neural correlate of the learning that odors have different associations. At this stage, both Go and NoGo cues were expected to carry salience, as they were both predictive of the outcome, regardless of whether it was a reward or penalty. In addition, no differentiation in their salience was expected since their different associations had only been recently learned. The third milestone was when rats showed a clear behavioral distinction between Go and NoGo cues, quantified as when the correct rate first reached 90%, meaning that they could assign higher salience to the high-valued Go cue compared to the low-valued NoGo cue.

We defined five learning phases relative to these three milestones. Phase 1 comprised the first 10 trials immediately following the first milestone, which was initial odor exposure. In this phase, DA responses were similar regardless of cue identity, consistent with the idea that the specific cue-outcome associations had not yet been established (left panel, fig. 2C. paired two-tailed t-test, p=0.49). ACh responses were also statistically indistinguishable from zero for both Go and NoGo cues as expected (right panel, fig. 2C. one sample t-test, p=0.66 and 0.63 for Go and NoGo trials, respectively). Phases 2 and 3 included the 10 trials immediately preceding and following the second milestone, which was the emergence of DA selectivity. In phase 2, significant ACh dips to both cues emerged (p=9.2e-6 and 8.1e-4 for Go and NoGo trials, respectively), but with no significant difference between them (p=0.27). In phase 3, although DA responses discriminated between Go and NoGo cues, ACh dips still did not show significant cue selectivity as expected (p=0.37), suggesting that DA differentiated cues preceding ACh. Phases 4 and 5 included the 10 trials immediately preceding and following the third milestone, when rats reached behavioral proficiency. In both phases, we found that ACh dips showed significant cue selectivity (p=7.5e-6 and 3.9e-4, respectively). These findings were consistent with our hypothesis that the dip in ACh encoded the salience of cues. Representative ACh and DA PSTHs in different stages are shown in fig. S2.

To further quantify the temporal progression of cue-evoked ACh and DA responses, we analyzed when ACh responses deviated from baseline and when ACh and DA responses differentiated between Go and NoGo cues over successive 10-trial bins. Our results revealed a sequence of emergence: cue-evoked ACh dips, defined as a significant drop below zero, appeared first at approximately 20.54 ± 4.78 trials (mean +/− S.E.M. across rats), followed by DA selectivity, defined as differential responses to Go versus NoGo cues, at 53.92 ± 9.06 trials (p=4.5e-3, two-tailed t-test; fig. 2D). ACh selectivity to cues emerged significantly later, at 95.69 ± 9.52 trials (p=5.2e-7 and 5.5e-3 compared to ACh dip and DA selectivity, respectively), and the behavioral criterion was reached last, at 248 ± 31.80 trials (p=4.8e-7, 8.0e-6 and 1.8e-4 compared to ACh dip, DA selectivity and ACh selectivity, respectively).

This sequential change highlights a structured progression during learning: ACh dips emerge early, potentially signaling initial attentional engagement with the cues. DA selectivity follows, marking the differentiation of cue-outcome associations. ACh selectivity appears after, indicating a refined salience assignment to specific cues, and ultimately, stable behavioral learning is achieved. These findings suggest that ACh may contribute to salience encoding, complementary to the function of DA in learning cue-specific associations. Further, the delayed emergence of ACh selectivity supports the idea that ACh is not directly involved in value discrimination. This temporal hierarchy provides insight into how cholinergic and dopaminergic systems evolved dynamically during learning.

### ACh dynamics and selectivity in the Go/NoGo task were captured by a hybrid attentional associative learning model.

**Fig. 3.**
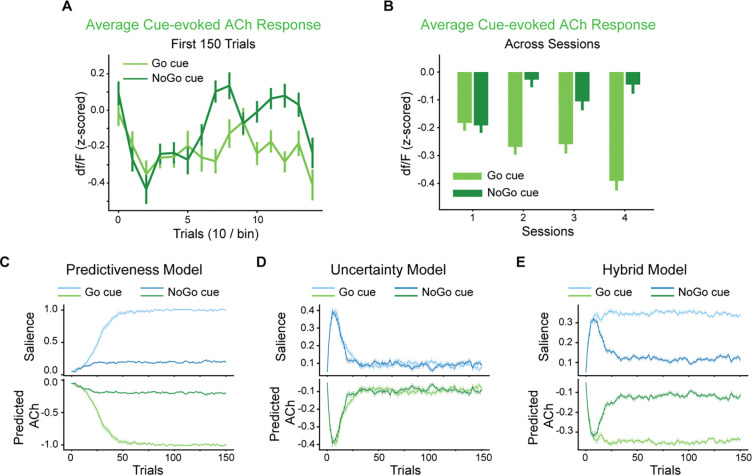
A hybrid attentional associative learning model captures biphasic dynamics and cue selectivity of the ACh responses. (**A**) Average cue-evoked ACh responses for Go (light green) and NoGo (dark green) cues in the first 150 trials. (**B**) Averaged cue-evoked ACh responses for Go (light green) and NoGo (dark green) cues across training sessions. The error bars indicate S.E.M. across trials. (**C-E**) The upper panels show the dynamics of salience predicted by the predictiveness model (C), based on the Mackintosh model; the uncertainty model, based on the Pearce-Hall model (D); and the hybrid model (E), which integrates salience mechanisms from both models. The lower panels show the ACh signal predicted by the models. The light lines indicate the Go cue, while the dark lines indicate the NoGo cue. The error bars indicate S.E.M. across runs.

To assess more quantitatively whether the ACh dips are consistent with salience signaling, we first analyzed ACh dynamics and then compared them to the normative salience terms defined by different attentional associative learning models. We initially examined the cue-evoked ACh responses for each odor cue condition over the first 150 trials. A biphasic trend was observed for the NoGo cue: the magnitude of ACh dips initially increased, followed by a subsequent decrease (fig. 3A). This decreasing trend held when we averaged the responses per session, with the NoGo cue-evoked ACh dips significantly less pronounced in the subsequent sessions compared to the first session (p=2.0e-5, 0.038, and 6.2e-4 compared to the 2^nd^/3^rd^/4^th^ sessions, respectively, fig. 3B). In contrast, ACh dips to the Go cue gradually increased in magnitude over learning and showed little evidence of subsequent decline (fig. 3 A and B), resulting in consistently stronger dips compared to the NoGo cue. We also analyzed the dopamine responses in the same way, and the results (fig. S3) were consistent with the prediction of the reward prediction error dynamics.

Subsequently, we reconstructed the task using computational learning models derived from the attentional associative learning framework. In this framework, salience modulates the learning rate and, together with prediction errors, adjusts stimulus-outcome associations. The precise definition of salience varies across different models, including the classical Mackintosh and Pearce-Hall models, and leads to different predictions. Mackintosh’s model assigns high salience to cues that are more predictive of outcomes than others, referred to as predictiveness-driven salience (PDS). The predictiveness-driven salience is further modulated by the absolute strength of the outcome, reflecting the fact that cues predicting higher reward value or stronger punishment retain greater salience. In contrast, the Pearce-Hall model defines salience as the mean absolute value of the RPE over time, reflecting the uncertainty of the cue in predicting outcomes. This uncertainty-driven salience (UDS) reflects the fact that animals allocate more attention to and learn faster about cues with uncertain outcomes. We built models (see Methods for details of all models) grounded in these two theoretical accounts and performed 20 runs for each to ensure robustness. As ACh signals dipped in response to cues, we inverted the sign of the salience predicted by these models and compared it with the ACh response.

Grounded in Mackintosh, the predictiveness model predicts an initial increase in salience for both Go and NoGo cues since they were the most reliable predictors among all perceivable stimuli (fig. 3C). The predictiveness model also distinguished between the two cues by assigning higher boundary for the salience of the Go cue, which was associated with a more motivationally salient outcome, i.e., water reward, compared to the NoGo cue’s outcome, i.e., reward omission or timeout penalty. However, the predictiveness model exhibited a significant limitation: it predicted a monotonic increase in salience, which implied a corresponding monotonic increase in the ACh dips (fig. 3C). This was inconsistent with the observed biphasic trend (fig. 3A), suggesting a critical shortcoming in the predictiveness model to capture the ACh dynamic.

An uncertainty model, derived from Pearce-Hall, was also developed to capture the eeffect of outcome uncertainty on cue salience. Initially, the uncertainty model did not learn the associations between presented cues and outcomes. Thus, a large RPE occurred upon outcome representation and enhanced cue salience. As learning progressed, the model learned the associations, eventually predicting reward outcomes accurately. As a result, salience for both Go and NoGo cues increased and subsequently decreased (fig. 3D). However, the uncertainty model predicted an identical salience pattern for Go and NoGo cues and failed to differentiate them (fig. 3A). Therefore, the uncertainty model was unable to capture the ACh selectivity in cue identity during learning.

Finally, we constructed a hybrid model following the framework proposed by Le Pelley et al (2004), which integrates mechanisms from both the Mackintosh and Pearce-Hall models of salience. In this model, the salience was defined as the weighted sum of the PDS from the Mackintosh model and the UDS from the Pearce-Hall model. We found that the UDS component in the hybrid model showed a biphasic pattern during learning, similar to the pure uncertainty model. Specifically, the UDS initially increased with the predicted uncertainty when cues were newly introduced, then subsequently decreased as the cue-outcome associations were learned. In parallel, the PDS component increased and was modulated by the learned value of cues, mirroring the predictiveness model. As a result, the salience signal in the hybrid model captured both the biphasic dynamics and cue selectivity of ACh dips (fig. 3 A and E).

To rule out the possibility that the distinct patterns of salience described for the models above were due to the arbitrarily chosen parameters, we fitted models by minimizing the mean squared error in reproducing ACh responses with rat behavioral data. This optimization was performed using differential evolution. Due to the simulation’s complexity, we fitted learning rates for the association strength, PDS and UDS, which we believe most significantly affect the prediction error. The results revealed a significantly smaller mean square error of reproducing the ACh responses by the hybrid model compared to the predictiveness model (p=4.1e-59, two-tailed t-test) and the uncertainty model (p=4.4e-56). Thus, the hybrid model comprehensively explained the observed ACh response patterns, indicating that ACh responses encode the combined predictiveness-driven and uncertainty-driven salience as proposed in Mackintosh and Pearce-Hall models.

### ACh correlates with dopamine response updates and changes in behavior.

Although the dynamics of cue-evoked ACh responses have been shown to align with predicted salience, the quantitative relationship between ACh dips and learning rate remained unestablished. To examine this, we asked whether the magnitude of ACh dips was associated with the rate at which outcome-evoked prediction errors updated cue value and behavioral choices.

**Fig. 4.**
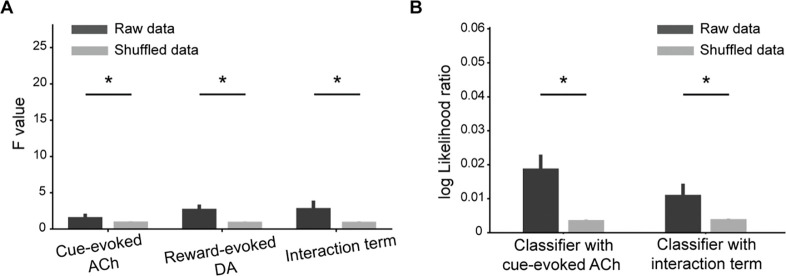
Ach responses modulate the dopamine and behavior updates. (**A**) F-values from the Granger causality test for the actual data (dark grey bars) versus shuffle data (light grey bars). Significant effects were found for reward-evoked DA responses (p=4.5e-8), its interaction with cue-evoked ACh responses (p=1.3e-6) on the subsequent cue-evoked DA responses, and the cue-evoked ACh responses (p=0.039). (**B**) The log-likelihood ratio of logistic classifiers that included either ACh (ACh model) or its interaction with DA against a baseline classifier using only reward-evoked DA. Each box plot shows the distribution of log-likelihood ratio values for actual data (dark grey) and for shuffled permutations (light grey). In both classifiers, the actual-data log-likelihood ratio is significantly higher than the shuffled distribution (p=7.9e-18 for ACh; p=4.0e-5 for interaction). The error bars indicate S.E.M. across rat. Each * indicates p<0.05 in a two-tailed t-test.

Given the role of DA signals in representing RPE, we used reward-evoked DA responses to estimate the reward-evoked prediction error, and changes in cue-evoked DA responses across trials to estimate cue value updates. To test whether the ACh dips correlate with the rate of cue value updating, we performed vector autoregression analyses in which four sets of predictors, i.e., cue-evoked ACh dips, reward-evoked DA response, their interaction term and the changes in cue-evoked DA from the previous $N_{\mathrm{lag}}$ trials, were used to predict estimated cue value updates. The lag length, $N_{\mathrm{lag}}=1$, was determined in advance using Bayesian information criteria (fig. S4A). Using Granger causality tests, we observed a significant effect of reward-evoked DA on cue value updates compared to shuffled controls (two-tailed t-test, p=4.5e-8, fig. 4A), consistent with the role of DA in representing RPE. Importantly, a strong and significant effect of the interaction between reward-evoked DA and cue-evoked ACh responses on cue value updates was also detected (p=1.3e-6, fig. 4A), confirming that ACh dips modulated the cue value updating.

Next, we measured whether the ACh dips were associated with behavioral changes in the NoGo cue. Trials with the Go cue were excluded, as rats consistently responded to it throughout learning, leading to little variability. We first trained a logistic classifier to predict the behavior only based on the reward-evoked prediction error from the previous trial, also estimated by the reward-evoked DA response. Next, we constructed two extended classifiers, one including the cue-evoked ACh dips and another including the interaction term as additional predictors. The contribution of each predictor was evaluated by the log-likelihood ratios (logLR) between extended classifiers and the basic one. To evaluate the significance, we shuffled the cue-evoked ACh dips and interaction term 100 times and recalculated the logLR for each shuffle. In both cases, classifiers with actual data showed significantly higher logLR compared to the shuffled controls, indicating that the ACh signal (p=7.9e-18, two-tailed t-test, fig. 4B) and its interaction with prediction error (p=4.0e-5) significantly contributed to behavioral predictions. These findings demonstrate that the cue-evoked ACh dips significantly modulate the rate of cue value updating and behavioral adaptation, supporting the hypothesis that ACh responses encode the salience of cues and modulate the learning rate.

### ACh dynamics reflect cue-related salience changes after reward contingency reversal.

Experiment 1 and accompanying simulations demonstrated that the dynamics of cue-evoked ACh dips in a stationary environment followed the predicted salience signal. In Experiment 2a, we aimed to extend our findings by testing if ACh dips track salience signals in a dynamic environment where reward contingencies change over time. Previous studies have shown that the cue salience does not automatically update following changes in reward value[35, 36], and the salience of a cue can persist even after the value of its predicted outcome decreases[37]. According to this, we hypothesized that after reversal in a Go/NoGo task, the ACh dips to the previously rewarding cue would persist, while the dips to the previously non-rewarded cue would increase in magnitude due to its new association with reward.

**Fig. 5.**
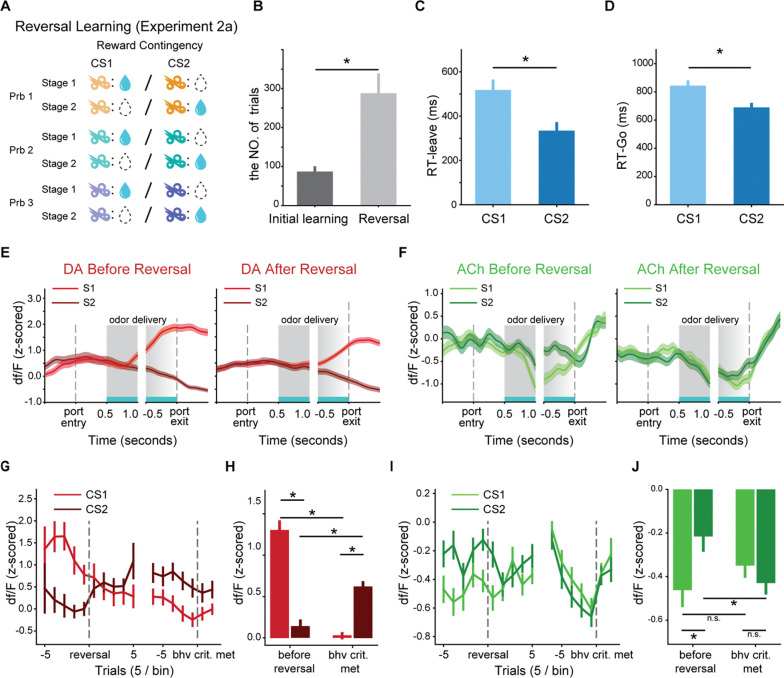
ACh responses to previously rewarded cue persisted after reversal (Experiment 2a). (**A**) Schematic of reward contingencies in Experiment 2a. The reversal learning task is composed of three problems, each with a different odor pair. The reward contingencies are reversed during the learning. (**B**) Number of trials required to reach the behavioral criterion during initial training (Stage 1) and after the reversal (Stage 2). (**C**) Latency to leave the odor port following CS1 or CS2 presentation. (**D**) Latency to enter the fluid well after CS1 or CS2. Rats exhibited shorter latencies for CS2 compared to CS1 after reversal. (**E**) Example DA dynamics before (left panel) and after reversal (right panel) within trials. (**F**) Example ACh dynamics before (left panel) and after reversal (right panel) within trials. (E-F) follow the same format as fig. 2A, respectively (**G**) Dynamics of the DA responses around the reversal point and upon reaching the reversal learning criterion are shown for CS1 and CS2. (**H**) Average DA responses to cues before the reversal and upon meeting the post-reversal behavioral criterion. (**I** and **J**). ACh signal dynamics and average responses. These panels follow the same format as panels (E and F), respectively. * in (B-D, F, and H) indicates p<0.05 in a two-tailed t-test.

To test these predictions, we conducted Experiment 2a, which consisted of a series of three reversal problems (fig. 5A), each involving two distinct stages. In stage 1, conditional stimulus 1 (CS1) was rewarded, while conditional stimulus 2 (CS2) was non-rewarded. Training continued for an additional 20 trials after rats first achieved the behavioral criterion, after which we initiated stage 2, in which we reversed the reward contingencies associated with CS1 and CS2. Training with these reversed contingencies continued across multiple sessions until rats met the same behavioral criterion after reversal. After successfully completing the first reversal task, we repeated this two-stage process with a second and third set of odor pairs.

Rats reached the post-reversal behavioral criterion with training, but required more trials than the initial reward contingency learning (fig. 5B; 272.6 ± 44.8 vs. 95.3 ± 15.3 trials, respectively; paired two-tailed t-test, p=0.0011). After reversals, rats exhibited shorter response latencies in both leaving the odor port (fig. 5B, p=5.8e-4) and entering the fluid well (fig. 5D, p=5.1e-5) following CS2 compared to CS1. These behavioral changes suggested that after reversal, rats associated both a higher value and greater motivation with CS2.

To gain an overall view of how DA and ACh signals changed dynamically during reversal learning, we plotted the PSTHs of DA and ACh signals from a representative rat, averaging across trials after the behavioral criterion was achieved both before and after reversal. Before reversal, DA responses were higher for CS1 compared to CS2, and this pattern reversed after reaching the post-reversal criterion (fig. 5E). In contrast, ACh showed a different pattern, initially exhibiting larger dips to CS1 before reversal. However, after the behavioral criterion was met, ACh dips to CS1 persisted and the ACh dips to CS2 were enhanced (fig. 5F).

To further quantify these dynamic changes across all rats, we averaged these signals around the reversal point and upon reaching the reversal learning criterion. The dynamics of the DA response remained consistent with RPEs. As expected, before reversals, the cue-evoked DA response to CS1 was significantly larger than that to CS2 (p=6.2e-13, left side of fig. 5 G and H). After meeting the post-reversal criterion, DA responses to CS2 increased (p=1.1e-5) and the responses to CS1 decreased compared to pre-reversal levels (p=9.9e-23, fig. 5 G and H). As a result, the DA responses to CS2 became significantly higher than CS1 (right side of fig. 5 G and H, p=1.1e-12).

We applied the same analysis to ACh responses. Consistent with Experiment 1, before reversals, both odors suppressed the ACh signal, with CS1 inducing a more pronounced dip compared to CS2 (left side of fig. 5I, p=0.02). After the reversal, the magnitude of the ACh dip evoked by CS2 increased while the dip evoked by CS1 persisted. In contrast to the DA responses, when rats reached the new criterion, ACh dips to CS2 increased (p=0.01), while the ACh dips to CS1 showed a slightly non-significant decrease (p=0.23, fig. 5 I and J). Consequently, ACh dips to CS1 and CS2 were statistically indistinguishable (right side of fig. 5 I and J, p=0.30). These results support our hypothesis that the ACh dips to the previously rewarding cue (CS1) remained for an extended period while dips to the newly rewarding cue (CS2) were enhanced, and are consistent with the view that ACh dips track a salience signal in dynamic environments and update more slowly than the value.

**Fig. 6.**
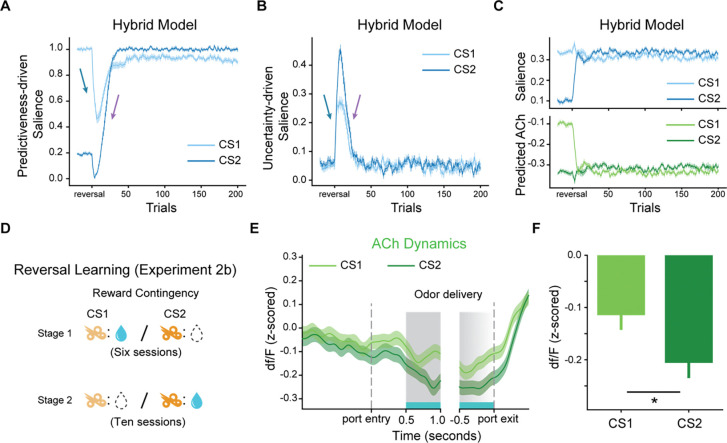
The hybrid associative learning model captured the dynamics of ACh signal in the reversal learning task (Experiment 2b). (**A-C**). Dynamics of predictiveness-driven salience (A), uncertainty-driven salience (B), total salience, and ACh responses predicted (upper and lower penal in (C), respectively) by the hybrid associative learning model following the reversal of reward contingencies. The light and dark lines represent signals in the CS1 and CS2 trials, respectively. Initially, reversal leads to elevated RPEs, leading to increases in UDS and decreases in PDS (blue arrows). With learning, the stimuli regain PDS due to improved relative predictiveness and lose UDS as uncertainty declines (purple arrows). The PDS and UDS changes of CS1 largely cancel out, while the new reward-associated CS2 shows a net increase in total salience due to enhanced UDS following the value update. The error bars indicate S.E.M. across runs. (**D**) Schematic of reward contingencies in Experiment 2b. (**E**) Empirical data showing the ACh dynamics within trials after extended post-reversal learning. (**F**) Averaged cue-evoked ACh responses across all rats in the last three sessions, showing the reversed cue selectivity in ACh signaling. * indicates p<0.05 in a two-tailed t-test.

Our hybrid associative learning model also successfully captured the characteristics of the ACh dips in the reversal learning task. After reversals, all odor cues predicted the opposite reward outcome, leading to elevated RPEs and diminished predictiveness. Consequently, there was an increase in UDS and a decrease in PDS (blue arrows in fig. 6, A and B). Once the values were updated, the stimuli regained PDS due to enhanced predictiveness and lost UDS owing to reduced uncertainty (purple arrows in fig. 6, A and B). For CS1, the changes in PDS and UDS largely canceled each other, leading to little change in the overall salience (light lines in fig. 6C). In contrast, CS2’s low pre-reversal PDS and its new association with water reward caused an increase in PDS, leading to larger total salience (dark lines in fig. 6C).

The salience of CS1 remained high when the post-reversal behavioral criterion was met due to the slow decline in the upper boundary of PDS, reflecting the persistent influence of reward history. However, the model does predict that, with extended training, this boundary would eventually decrease, causing low PDS and reduced total salience for CS1. Thus, we hypothesized that extended post-reversal training would invert the preference of ACh dips in cues. To test this, we conducted Experiment 2b (fig. 6D). In this experiment, five rats were trained in a Go/NoGo task for six sessions, and from the seventh session, we reversed the reward contingencies 20 trials after the rats achieved the behavioral criterion. Training with the reversed contingencies continued for ten sessions. Pooling the data from the final three sessions, we observed a reversed selectivity in ACh dips. CS2 evoked significantly more pronounced ACh dips than CS1 (p=0.025, fig. 6 E and F). These results support the role of ACh dips in encoding cue salience and demonstrate that their dynamics adapt to the changing reward contingencies over longer timescales.

## Discussion

We explored the role of ACh in the learning process by recording ACh and DA signals in the NAcc across multiple experiments. We found that cue-evoked ACh response reflects an acquired salience term that modulates the learning rate and that can be captured by a hybrid attentional associative learning model that integrates salience driven by both predictiveness and uncertainty. Initially, the ACh responses did not change with presented novel cues, as these cues had not yet gained any salience. However, as cues acquired salience through predictiveness and uncertainty, characteristic ACh dips in response to cues emerged. The magnitude of these dips evolved over learning and reflected the changing salience across different learning phases. In addition, the cue-evoked ACh dips persisted even when the value of cues decreased, demonstrating that these dips tracked the overall relevance of the cues, and not the value itself.

While traditional reinforcement learning theories often assume a constant learning rate, effective learning requires adjusting this rate based on environmental volatility[38, 39]. Recent studies suggest that dopamine transients encode RPEs independently of learning rates[40], raising the question of how the learning rate is regulated. Previous studies have suggested that the cholinergic system is well-suited for this function. Extensive interactions between dopaminergic and cholinergic systems have been observed in multiple brain regions[25, 41], and ACh dips in the NAcc have been proposed to create a permissive window for phasic dopamine increases and facilitate synaptic plasticity[32, 42], thus supporting the hypothesis that the ACh dips signal the learning rate. Inspired by these findings, we directly demonstrated that ACh in NAcc followed the salience changes predicted by a hybrid attentional associative learning model and affected the rate of DA and behavior updates, which was aligned with the role of salience in regulating the learning rate. These results suggest a mechanism of how the learning rate is modulated in the brain and highlight ACh’s role in adaptive learning.

Although the accumbal cholinergic system has long been known to play a crucial role in learning, its precise computational role has remained unspecified. Our study suggests that cue-evoked ACh dips encode cue salience, which explains not only our recording results but also previous manipulation findings. For instance, mice with a selective knockout of the vesicular acetylcholine transporter (VAChTcKO) failed to discriminate between cues paired with reward (CS+) and non-reward (CS−) during acquisition sessions[31]. In addition, significant ACh dips to the CS+, observed in control mice, were absent in VAChTcKO mice[31]. These results also suggest that ACh dips encode salience and regulate the learning rate. Without these dips, VAChTcKO mice could not assign appropriate salience to the cues, resulting in slower learning and a failure to learn the reward contingencies as control mice.

This proposal contrasts with previous studies that have concluded that ACh signals in the dorsal striatum[43] and basal forebrain[44] encode RPEs. While the ACh responses in NAcc are also selective to cue value and decrease in response to reward outcomes as learning progresses, similar to an RPE signal, we believe that ACh in the NAcc does not directly encode RPEs as in the basal forebrain. First, during the initial learning phase, ACh responses are not modulated by cues. With continued training, however, ACh becomes progressively inhibited by both Go and NoGo cues, even though these cues are associated with opposite RPEs. Second, DA signals, known to encode RPEs, develop cue selectivity earlier than the ACh does. Especially in the reversal learning task, the ACh responses could not differentiate cues even when rats learned the reversed reward contingency without further training. These observations suggest that ACh selectivity may be tied to more computationally complex variables like salience, rather than directly encoding RPE or value.

Importantly, the correspondence between cue-evoked ACh dips and the concept of cue salience does not rely on the specific form of the hybrid model used in this study. A similar model proposed by Esber and Haselgrove (2011)[6], which also integrates PDS and UDS, produces similar predictions. Unlike the Mackintosh and Pearce-Hall models, this model defines PDS as the sum of a cue’s association strength with positive and negative reinforcers, and UDS reflects the extent to which preceding events predict the cue itself. Total salience is the difference between these two components (Supp information). In the Go/NoGo task, the PDS increased as cue-outcome associations were learned, and the UDS increased with repeated cue presentations. With an appropriate learning rate, total salience would initially increase and subsequently decline. Since the water reward supports stronger associations, the Go cue exhibited higher salience, consistent with predictions from our hybrid model (fig. S5). This robustness suggests that the salience carried by ACh reflects a more general principle of learning, transcending the particular model adopted here.

The associative learning model we used in the current study proposes that acquired salience can modulate the learning rate and subsequent RPEs, while the RPEs are quantitatively related to the prediction uncertainty and can, in turn, affect salience. Such a mechanism highlights potential interactions between cholinergic and dopaminergic systems across trials. Supporting this, we observed significant effects of cue-evoked ACh dips on the update of DA and behaviors in the current study. However, we failed to observe a reciprocal effect of the DA signal on the ACh (fig. S4 B and C), possibly due to the complexity of salience dynamics, which may obscure the effects of individual factors. The neural mechanisms underlying this modulation remain unclear and require further investigation.

In summary, our findings revealed that the cue-evoked ACh dips in NAcc encode the associative or acquired salience of stimuli across various learning stages and tasks. We found surprising consistency between the ACh dips and the salience term predicted by a hybrid attentional associative learning model, where associative salience was determined by both predictiveness-driven and uncertainty-driven salience. These findings, together with the observed influence of ACh on DA and behavior updates, support ACh’s role in encoding salience and modulating learning rate throughout the learning process.

## Materials and Methods

### Experimental model and subject details

We included 22 male Long–Evans rats weighing over 450g at the start of the experiment, sourced from Charles River Laboratories and the NIDA IRP Breeding Facility. These rats were housed on a 12-h light-dark cycle at 25°C. They had free access to food but were water-restricted to approximately 85% of their original weight during the experiments. All rats received around 20 ml of water per day. Behavioral testing was performed during the light phase of the light-dark schedule. Experiment 1 was conducted on fourteen rats. One rat was excluded from the analyses due to markedly different ACh signals, although including this rat did not alter the overall conclusions. The ACh data for this rat, as well as the results for all fourteen rats combined, were provided in the supplementary materials (fig. S6). Of the fourteen rats, five were subsequently used for the long-term reversal learning study (Experiment 2b). An additional eight rats were initially used in Experiment 3, with five of them later also involved in Experiment 2a. One rat was not trained for Experiment 2a due to health issues, while two rats failed to meet the behavioral criterion in certain reversal problems and were therefore excluded from analyses. All procedures followed the National Institutes of Health guidelines, as determined by the National Institute on Drug Abuse Intramural Research Program (NIDA IRP) Animal Care and Use Committee (protocol no. 23-CNRB-108).

### Surgical procedures

Animals were anesthetized with isoflurane (3–5% for induction and 1–2% for maintenance). To simultaneously measure the dopamine and acetylcholine release in the nucleus accumbens core, we injected the D1 receptor-based red dopamine sensor-transfecting virus (pAAV-hsyn-rDA3m) and the muscarinic M3 receptor-based green acetylcholine sensor-transfecting virus (AAV-hSyn-gAch4h) into the unilateral NAcc of rats. The injection coordinates were AP +1.7 mm, ML ±1.7 mm, and DV −6.3 and −6.2 mm from the brain surface. Rats injected in the left and right brain were counterbalanced. In the five rats used for both Experiment 1 and the long-term reversal learning task, the DA and ACh sensor-transfecting viruses were injected into different hemispheres. For all other rats, both sensors were injected into the same hemisphere. A total of 1.0 μL of dopamine and acetylcholine sensor-transfecting virus, 0.5 μL for each, was delivered in each site at 0.1 μL/min via an infusion pump. All viruses were obtained from BrainVTA. The injection needle was held in for an additional 5 min after the completion of injection to avoid the backflow of viruses. An optic fiber (0.37 NA, 200μm diameter, Neurophotometrics, CA) was implanted in the most dorsal viral infusion location. Exposed fiber ferrules and a protective black 3D-printed headcap were secured to the skull with dental cement. After surgery, rats were given Cephalexin (15 mg/kg orally, once daily) for two weeks to prevent infections. Training started at least four weeks after the surgery for the virus expression.

### Fiber Photometry

#### Recording

Fluorescence signals were recorded using custom-ordered multi-pronged fiber optic patch cables (200 μm diameter, 0.37 NA, Doric Lenses, Canada) that were attached to the optic fiber ferrules on the skull of the rats with brass sleeves (Thorlabs, NJ). Up to 2 fibers were connected at a time in each rat for recordings, and they were shielded and secured with a custom 3D-printed headcap-swivel shielding system that allowed for the relatively free movement of the rats without the use of optic commutators and prevented the spillover of light during recordings.

Recordings were conducted using an FP3002 system (Neurophotometrics, CA), by providing fiber-coupled LEDs at 470nm (green, active acetylcholine-dependent signal), 560nm (red, active dopamine-dependent signal) and 415 nm (isosbestic reference signal) excitation light through the patch cord in interleaved LED pulses at 150 Hz (50 Hz acquisition rate for each channel). The light was reflected through a dichroic mirror and onto a 20×Olympus objective. Excitation power was measured at ~150–200 μW at the tip of the patch cord. Emitted fluorescent light was captured through the same cords, separated with an image splitting filter, and captured via a high quantum efficiency CMOS camera. Signals were acquired and synchronized with behavioral events using Bonsai.

#### Pre-processing

We filtered the raw fluorescence signals from the 470 nm (active), 560 nm (active), and 415 nm (reference) channels by using a causal median filter and a second-order Butterworth low-pass filter with a cutoff frequency of 5 Hz. Next, we fitted each channel’s data with a second-order Butterworth high-pass filter with a cutoff frequency of 0.002 Hz, aiming to eliminate the exponential decay in fluorescence caused by factors like photobleaching. After that, the reference channel data was then fitted to each active signal using second-order polynomial regressions, and the fitted data was subsequently subtracted from the active channel and divided by the exponential fit of the active channel to remove signal-independent variations in fluorescence. Finally, the resulting active signal was z-scored for each session.

### Behavioral task:

Rats were trained and recorded at least 4 weeks after the surgeries in standard operant boxes, which were aluminum chambers approximately 18 inches long in height, depth, and width. A central odor port was located above the two fluid wells located on the left wall of the chamber, and one well was physically blocked and not used during this study. Two lights were located above the odor port. The odor port was connected to an airflow dilution olfactometer to allow the rapid delivery and removal of odor cues. Odors were chosen from compounds obtained from International Flavors and Fragrances (New York, NY). Odor delivery and fluid delivery to the odor port and fluid well were controlled by the behavioral computer via a system of flow meters and solenoids. The entry into the odor port and fluid well was detected by the interruptions of an infrared beam, which was connected to the behavioral computer.

All experiments followed the same procedures in a single trial and differed only in the odor used and reward contingency. After the illumination of lights inside the box, rats were allowed to perform a nosepoke into the central odor port to initiate a new trial. Following a 500ms delay, an odor cue was presented for another 500ms. The presented odor was randomly selected from two possible cues that differed in different experiments and problems, and determined the reward availability in the fluid well. After the termination of odor delivery, the rats were free to leave the odor port and had 3 seconds to make a response at the fluid well. If a response was initiated, 0.05ml water was delivered 500ms later in reward trials, while no fluid was delivered in no-reward trials. During reward trials, the panel lights remained illuminated until the rat exited the fluid well, and the lights turned off indicated the end of a trial. In no-reward trials, the lights were turned off immediately after the response. If the rats did not respond within 3 seconds after leaving the odor port, the lights were extinguished then. Inter-trial intervals commenced upon the turnoff of the panel lights and lasted for 4 seconds, extending to 8 seconds following responses in non-rewarded trials. Before involving any experiment, rats were trained with no odor and received water rewards at the fluid well to familiarize themselves with the task procedures.

#### Experiment 1 (Go/NoGo task):

Fourteen naive rats were included in experiment 1 and trained with a Go/NoGo task using the task structure mentioned above. Two distinct odor cues were used: one “Go” cue associated with a reward and the other “NoGo” cue associated with no reward (fig. 1B). To keep an overall balance in the number of positive vs negative trials throughout the session, we included 25 positive and 25 negative trials in every 50 trial block, but the order was pseudo-randomly generated independently. Rats were trained to learn the associations between cues and outcomes for at least six sessions.

#### Experiment 2a (reversal task):

Seven naive rats were trained with three Go/NoGo problems and were then involved in Experiment 2a. Data from two were excluded from analyses since they failed to meet the behavioral criterion in certain reversal problems. Experiment 2a consisted of three reversal problems (fig. 5A), each involving two stages. In the first stage, the rats were trained with a Go/NoGo problem used in experiment 1. Once rats achieved the behavioral criterion on the initial problem, 18 correct responses within a 20-trial moving block, training was stopped 20 trials later to ensure that rats were equally proficient. The second stage was initiated once rats reached the criterion again in the next session. In the second stage, the reward contingencies between cues and outcomes are reversed, i.e., the cue previously associated with a reward now indicated no reward, and the cue previously associated with no reward led to a reward. To encourage rats to learn this reversal, we introduced three teaching trials after each reversal, in which the previously unrewarded cues were presented, and rats had unlimited time to respond at the fluid well. These teaching trials increased the probability that rats responded at the fluid well and discovered that the reward contingency was reversed. Training with reversed contingencies continued over multiple sessions until the rats met the same behavioral criterion. After the successful completion of the first reversal, the reward contingencies for the second and third sets of odor pairs were similarly trained and subsequently reversed.

#### Experiment 2b (reversal task with extensive training)

Five rats used in Experiment 1 were trained with a Go/NoGo task over six sessions. In the seventh session, we reversed the reward contingencies 20 trials after the rats achieved the behavioral criterion. Training with the reversed contingencies then continued for ten sessions.

### Histological procedures

After completion of the experiment, rats were perfused with chilled phosphate buffer saline (PBS) followed by 4% paraformaldehyde in PBS. The brains were then immersed in 20% sucrose in PBS for at least 24 hours and frozen. The brains were sliced at 50 μm in series for determination of optic fiber implant location in NAcc core and were then used for fluorescent immunohistochemistry. Brain slices were further stained with DAPI (Vectashield-DAPI, Vector Lab, Burlingame, CA), and processed for immunohistochemical detection of green and red fluorescent protein. For fluorescence immunohistochemistry, gACh4h was immunostained using a chicken anti-GFP antibody (1:1,000, Abcam, catalog #ab13970) in 0.1% Triton X-100/1× PBS and incubated in Alexa Fluor 488 AffiniPure Donkey Anti-Chicken IgY (IgG) secondary antibody (H+L, code: 703–545-155, RRID: AB_2340375, dilution: 1:100) overnight. rDA3m was immunostained using a rabbit anti-RFP antibody (1:1,000, Rockland, catalog #600–401-379) in 0.1% Triton X-100/1× PBS and followed by incubated in Alexa Fluor 594 AffiniPure Donkey Anti-Rabbit IgG secondary antibody (H+L, code: 711–585-152, RRID: AB_2340621, dilution 1:100). Fluorescent microscopy images of the slides were acquired with a BZ-X800 Keyence microscope. Expression patterns were extracted from the images and then superimposed on anatomical templates.

### Vector autoregression modeling and Granger causality testing

The hypothesis that the level of ACh release evoked by a cue modulates the learning rat led to the prediction that the ACh release, in combination with the prediction error induced by the reward outcome, could significantly influence the updating of the cue’s value. Prediction error evoked by the reward outcome can be estimated by the corresponding DA response, while the cue’s value updates can be inferred from changes in the cue-evoked DA response across trials.


$$DA_{\mathrm{cue}}^{t+1}-DA_{\mathrm{cue}}^{t}\sim \left(ACh_{t}^{\mathrm{cue}}+\;{\mathrm{baseine}}_{1}\right)*(DA_{t}^{\mathrm{reward}}+\;{\mathrm{baseline}}_{2})$$

To test this relationship, we applied a vector autoregression model separately to all trials for each condition for each condition separately. This analysis assessed the correlation between the cue-evoked DA response, the cue-evoked ACh response, the reward-evoked DA response, and their interaction term. The model is expressed as:

$$DA_{\mathrm{cue}}^{t+1}-DA_{\mathrm{cue}}^{t}=c+\sum_{n=0}^{lag}k_{1}*\left(DA_{\mathrm{cue}}^{t-n}-DA_{\mathrm{cue}}^{t-n-1}\right)+k_{2}*ACh_{\mathrm{cue}}^{t-n}+k_{3}*DA_{\mathrm{reward}}^{t-n}+\;k_{4}*DA_{\mathrm{reward}}^{t-n}*ACh_{\mathrm{cue}}^{t-n}+\varepsilon ,$$

where c is a constant, $k_{i}$ are model parameters and ε is the error term. Bayesian information criterion (BIC) was calculated for different lags, and the lag with the lowest information criterion value was selected for further analysis (fig. S4a).

Next, the model was fitted separately for each session and trial type (presented odor). Granger causality analysis was then applied to determine whether the cue-evoked ACh response, reward-evoked DA response, and their interaction term provided significant information about the cue-evoked DA response beyond what could be explained by its past values. The F values were from the original data compared to those obtained from the control data, where the $DA_{\mathrm{cue}}$ were shuffled 1000 times, to determine the statistical significance of each factor’s effect on cue-evoked DA responses.

### Attentional associative learning models

We used three attentional associative learning models to simulate the salience dynamics of stimuli in the learning process. The mechanisms for updating associative strength were the same across models, differing only in how salience was defined. By varying the definition of salience, three models provide different predictions on the salience dynamics during learning.

The associative strength for a conditioned stimulus, A, was represented by an excitatory component, $V_{A}$, and an inhibitory component, ${\bar{V}}_{A}$, indicating the expectation of positive (water reward) and negative reinforcer (timeout), respectively. The net associative strength, $V_{A}^{\text{net}}$, is calculated as:

$$V_{A}^{\text{net}}=V_{A}-{\bar{V}}_{A}.$$

The updates to these components are driven by the prediction error, $\delta_{v}$, defined as:

$$\delta_{v}=\lambda -\Sigma_{X}V_{X}^{net},$$

where X represents the set of stimuli represented in the environment and λ is the signed maximum associative strength supported by the reinforcer, representing either the reward or penalty value. Specifically, λ is set to 1 for water reward, −0.1 for reward omission, and −0.2 for timeout penalty. The models update the excitatory and inhibitory components of the associative strength based on the sign and magnitude of the $\delta_{v}$,

$$\left\{V_{A}=V_{A}+\eta_{V}\cdot S_{A}\cdot \delta_{v}+N(0,0.025),\;\;\;\text{if}\;\delta_{v}>0\;{\bar{V}}_{A}={\bar{V}}_{A}+\eta_{V}\cdot S_{A}\cdot \delta_{v}+N(0,0.0.25),\;\text{if}\;\delta_{v}<0\right.$$

where $\eta_{V}$ is the learning rates for associative strength, $S_{A}$, indicating the salience of stimulus A, modulates the overall impact of the $\delta_{v}$ on the associative strength and N(0,0.025) is the Gaussian noise with mean 0 and variance 0.025.

#### The predictiveness model:

The predictiveness model is grounded in the Mackintosh model. The salience, S, is determined by the predictiveness, α, of a stimulus. Stimuli providing higher predictiveness compared to other perceivable stimuli are assigned higher salience. The change in α, $\delta_{\alpha }$, is defined as the difference between the absolute error for other stimuli (X≠A) and the absolute error for the target stimulus (A):

$$\delta_{\alpha }=|\lambda -\sum_{X\neq A}V_{X}^{\text{net}\;}|-|\lambda -V_{A}^{\text{net}\;}|$$

$$\alpha =\left\{\begin{array}{ll} \alpha +\eta_{\alpha }^{\text{pos}\;}\cdot \delta_{\alpha }+N(0,0.05),\; & \text{if}\;\delta_{\alpha }>0\\ \alpha +\eta_{\alpha }^{\text{neg}\;}\cdot \delta_{\alpha }+N(0,0.05),\; & \text{if}\;\delta_{\alpha }<0 \end{array}\right.$$

where $\eta_{\alpha }^{\text{pos}}$ and $\eta_{\alpha }^{\text{neg}}$ are the learning rates for increasing and decreasing α, respectively. To ensure that α remains within a meaningful range, it is rectified according to an upper limit, $\alpha_{\text{upper}}$, and a lower limit, $\alpha_{\text{lower}}$. If α exceeds the upper limit, it is set to the upper limit, and if it drops below the lower limit, it is set to the lower limit. $\alpha_{\text{lower}}$ is pre-defined, while $\alpha_{\text{upper}}$ is dynamic and determined by the absolute strength of the reinforcer.


$$\alpha_{\text{upper}\;}=\left\{\begin{array}{cc} 0.9995\cdot \alpha_{\text{upper}},\; & \text{if}\;|\lambda |<\alpha_{\text{upper}\;}\\ |\lambda |,\; & \text{if}\;|\lambda |>\alpha_{\text{upper}\;} \end{array}\right.$$

#### The uncertainty model:

The uncertainty model is grounded in the Pearce-Hall model. The salience of a stimulus is determined by its uncertainty. Stimuli, which provide lower predictiveness and indicate higher uncertainty about the upcoming observations, are assigned higher salience, σ. The error in σ, $\delta_{\sigma }$ is defined as:

$$\delta_{\sigma }=|\lambda -\sum_{X}V_{X}^{net}|$$

Then σ is then updated similarly to α, and the learning rate varies based on whether $\delta_{\sigma }$ is positive or negative:

$$\sigma =\left\{\begin{array}{ll} \left(1-\eta_{\sigma }^{pos}\right)\cdot \sigma +\eta_{\sigma }^{pos}\cdot \delta_{\sigma }+N(0,0.05),\; & \text{if}\;\delta_{\sigma }>0\\ \left(1-\eta_{\sigma }^{neg}\right)\cdot \sigma +\eta_{\sigma }^{neg}\cdot \delta_{\sigma }+N(0,0.05),\; & \text{if}\;\delta_{\sigma }<0 \end{array}\right.$$

#### The hybrid model:

The hybrid model integrates salience components from both the Mackintosh and Pearce-Hall models, The salience, S, is determined by the weighted sum of predictiveness-driven and uncertainty-driven salience of a stimulus.

S=ω⋅α+(1-ω)⋅σ

where 0<ω<1 determines the relative contribution of α and σ. In all simulations, ω is set to 0.3. The dynamics and update rules of α and σ are the same as those described in the predictiveness model and the uncertainty model.

#### Go/NoGo task

We simplified the task structure in our simulations. Completed trials from rats were used to train the models. For each trial, we assumed that two stimuli were represented: the presented odor and the background stimuli in the environment. Following these cues, one of three reinforcers was presented: water reward, reward omission, and timeout penalty. In all three models, $\eta_{V}=0.8$, $\eta_{\alpha }^{pos}=\eta_{\alpha }^{neg}=0.3$, and $\eta_{\sigma }^{pos}=\eta_{\sigma }^{neg}=0.5$.

To evaluate the goodness of fit for ACh signal in the Go/NoGo task, we used rat behavioral data and fitted models by minimizing the mean squared error in reproducing ACh responses. This optimization was performed using differential evolution. Due to the simulation’s complexity, we fitted three key free parameters in each model that we believe most significantly affected the results. For the predictiveness model, we relaxed the assumption that $\eta_{\alpha }^{pos}=\eta_{\alpha }^{neg}$ and set $\eta_{\sigma }^{pos}=\eta_{\sigma }^{neg}=0$. The fitted parameters were $\eta_{V}$, $\eta_{\alpha }^{\text{pos}}$ and $\eta_{\alpha }^{\text{neg}}$. For the uncertainty model, we relaxed the assumption that $\eta_{\sigma }^{\text{pos}\;}=\eta_{\sigma }^{\text{neg}\;}$, and set $\eta_{\alpha }^{\text{pos}}=\eta_{\alpha }^{\text{neg}}=0$. The fitted parameters were $\eta_{V}$, $\eta_{\sigma }^{\text{pos}}$ and $\eta_{\sigma }^{\text{neg}}$. For the hybrid model, we assumed $\eta_{\alpha }^{\text{pos}}=\eta_{\alpha }^{\text{neg}}=\eta_{\alpha }$ and $\eta_{\sigma }^{\text{pos}}=\eta_{\sigma }^{\text{neg}}=\eta_{\sigma }$. The fitted parameters were $\eta_{V}$, $\eta_{\alpha }$ and $\eta_{\sigma }$. We compared the losses from all entries in the population generated by the fitting algorithm, i.e., differential evolution, across different models to evaluate their relative fitting performance.

#### Reversal task

To capture the dynamics of the ACh signal in the reversal task, we relaxed the assumptions $\eta_{\alpha }^{\text{pos}}=\eta_{\alpha }^{\text{neg}}$ and $\eta_{\sigma }^{\text{pos}}=\eta_{\sigma }^{\text{neg}}$. The results shown in fig. 6 were obtained using the parameters: $\eta_{\alpha }^{\text{pos}}=0.3$ and $\eta_{\sigma }^{\text{pos}}=0.15$. All other parameters were consistent with those used in the Go/NoGo task.

## Supplementary Material

Supplement 1

Supplementary text and figures S1-S6.

## Data Availability

The data generated in this study will be deposited in the Zenodo database and accessible upon acceptance. All custom code used for reported analyses in this study will be available at GitHub upon acceptance.
