# Supplementary material for: Accumbal acetylcholine signals associative salience during learning
Source: bioRxiv. 2025 Apr 25:2025.01.06.631529. Originally published 2025 Jan 6. Preprint. [Version 2] doi: 10.1101/2025.01.06.631529 (PMC11741319; doi:10.1101/2025.01.06.631529)
Supplement: Supplement 1 [file media-1.pdf]

Science Advances
AAAS

Supplementary Materials for

Accumbal acetylcholine signals associative salience

Zhewei Zhang *et al.*

*Corresponding author. Email: zhewei.zhang@nih.gov, kaue.m.costa@gmail.com, and geoffrey.schoenbaum@nih.gov

This PDF file includes:

Supplementary Text
Figs. S1 to S7

Supplementary Text

<u>The attentional associative learning models based on Esber and Haselgrove (2011)</u>

Esber and Haselgrove (2011) is another influential hybrid attentional associative learning model (hereafter referred to as the E-H model). Here, we adopted the E-H model to reproduce the ACh responses we observed in the Go/No-Go task. The Go/No-GO task structure was formalized as we did for the other models in the main text.
Like the models discussed in the main text, the E-H model represents a conditioned stimulus's associative strength through two components: an excitatory component for positive reinforcer associations and an inhibitory component for negative reinforcer associations. The associative updating mechanism follows similar principles to the previously discussed models, a distinction lies in its updating pattern. The E-H model updates both positive and negative associations on every trial, rather than selectively updating positive associations only during trials with positive RPE and negative associations only during trials with negative RPE.
Similar to Model 3, the E-H model incorporates both predictiveness-driven and uncertainty-driven components in calculating salience. However, these two salience components are defined differently from previous models. In the E-H model, the predictive-drive salience, α, is defined as the sum of positive and negative association strength and we updated α in a temporal difference manner. This follows from the assumption that the cue will signal emotionally potent positive and negative reinforcers simultaneously, which have additive effects on salience:

$$\delta_\alpha = V_A + \bar{V}_A - \alpha$$

$$\alpha = (1 - \eta_\alpha) \cdot \alpha + \eta_\alpha \cdot \delta_\alpha + N(0, 0.05)$$

Unlike Le Pelly’s model, which focuses on outcome prediction uncertainty, the E-H model shifts the focus from outcome uncertainty to cue uncertainty, i.e., the uncertainty of the cue representation based on its context and preceding cues. Thus, the uncertainty-driven salience, σ, decreases as the cue becomes more predictable from contextual and preceding events.

$$\delta_\alpha = p - \sigma$$

$$\sigma = (1 - \eta_\sigma) \cdot \sigma + \eta_\sigma \cdot \delta_\alpha + N(0, 0.05)$$

where $p = 1$ when cue A is represented in the current trial. Otherwise, $p = 0$.

The E-H model determines a cue's overall salience, S, by calculating the difference between its predictiveness-driven and uncertainty-driven components, rather than their sum. This approach aligns with the model's assumption that a cue becomes less salient when it becomes predictable.

$$S = b + \alpha - \omega \cdot \sigma$$

where $\omega = 0.6$ determines the relative contribution of α and σ. $b = 0.2$ is the baseline for the salience. When we ran this model, $\eta_V = 0.4$, $\eta_\alpha = 0.5$, and $\eta_\sigma = 0.05$.

Fig. S1.

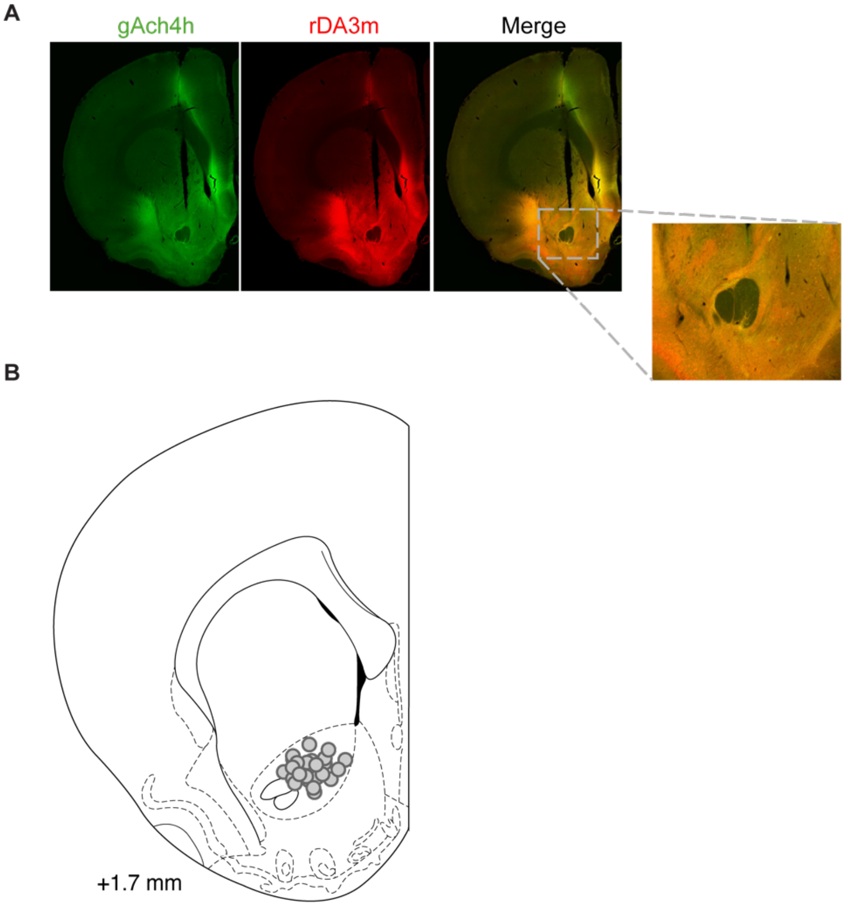

Fig. S1. **Histological verification and photometry recording locations.** (**A**) Representative histological microphotograph with histological detection of both sensors. (**B**) Location of fiber tips in the NAcc for all recorded rats (N=22 rats). Among these, 5 rats had fibers implanted in both hemispheres and 18 rats had fibers implanted in a one hemisphere.

Fig. S2.

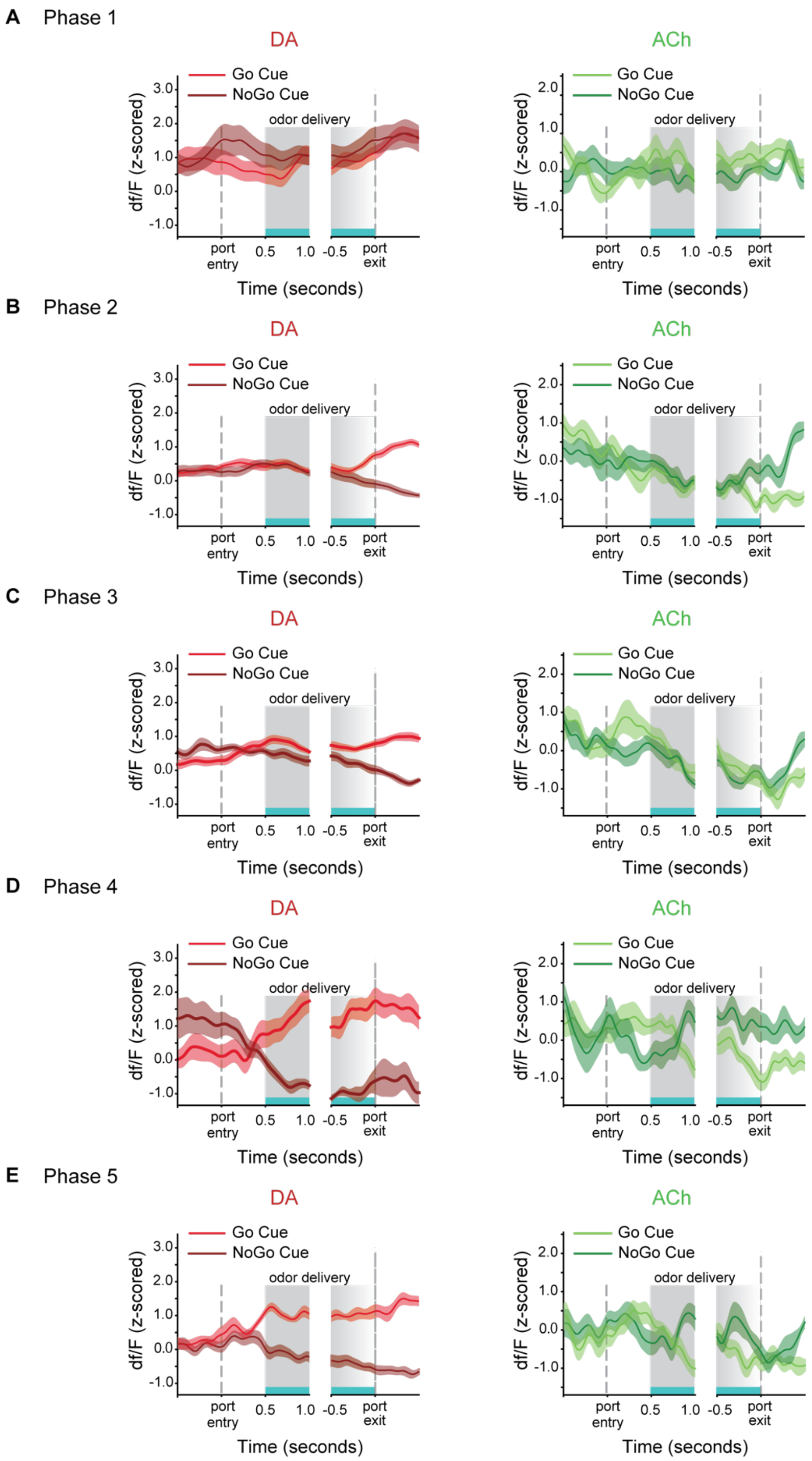
A Phase 1
DA
ACh
Go Cue
NoGo Cue
odor delivery
df/F (z-scored)
3.0
2.0
1.0
0.0
-1.0
port entry
0.5
1.0
-0.5
port exit
Time (seconds)
B Phase 2
DA
ACh
Go Cue
NoGo Cue
odor delivery
df/F (z-scored)
Time (seconds)
C Phase 3
DA
ACh
Go Cue
NoGo Cue
odor delivery
df/F (z-scored)
Time (seconds)
D Phase 4
DA
ACh
Go Cue
NoGo Cue
odor delivery
df/F (z-scored)
Time (seconds)
E Phase 5
DA
ACh
Go Cue
NoGo Cue
odor delivery
df/F (z-scored)
Time (seconds)

Fig. S2. Representative ACh and DA PSTHs from an example rat (Experiment 1) in different stages. (**A**) The first ten trials. (**B**) Ten trials immediately before and after DA selectivity emerged. (**C**) Ten trials immediately before and after behavioral criterion was met.

Fig. S3.

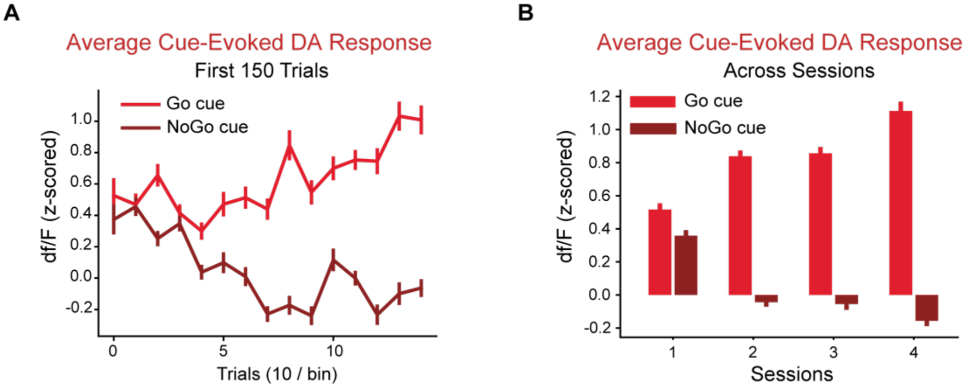

Fig. S3. Dynamic changes of dopamine response across trials and sessions. (**A**) Average cue-evoked DA responses for Go (light red) and NoGo (dark red) cues in the first 150 trials. (**B**) Averaged cue-evoked DA responses for Go (light red) and NoGo (dark red) cues across training sessions. The error bars indicate S.E.M. across trials.

Fig. S4.

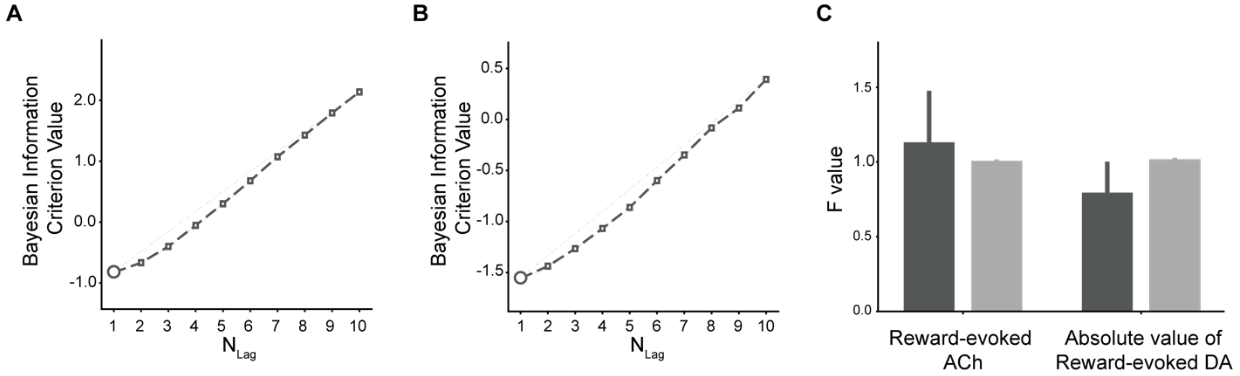

Fig. S4. Determination of VAR lag length and Predict cue-evoked ACh response with DA responses. (**A**) Bayesian Information Criterion (BIC) values for VAR models predicting cue value updates (fig. 3A) across different lag lengths. The circle indicates the lag (N_{lag} =1) with lowest BIC value. (**B**) BIC values for VAR models predicting cue-evoked ACh signals based on reward-evoked DA signals, their absolute values, and past cue-evoked ACh responses, with varying lag lengths. The circle indicates the lag (N_{lag} =1) with lowest BIC value. (**C**) F-values from the Granger causality test for the actual data (dark grey bars) versus shuffle data (light grey bars). No significant effects were found for reward-evoked DA signals (p-value=0.66) or their absolute value (p-value=0.45) on the subsequent cue-evoked ACh signal. The error bars in indicate S.E across trials.

Fig. S5.

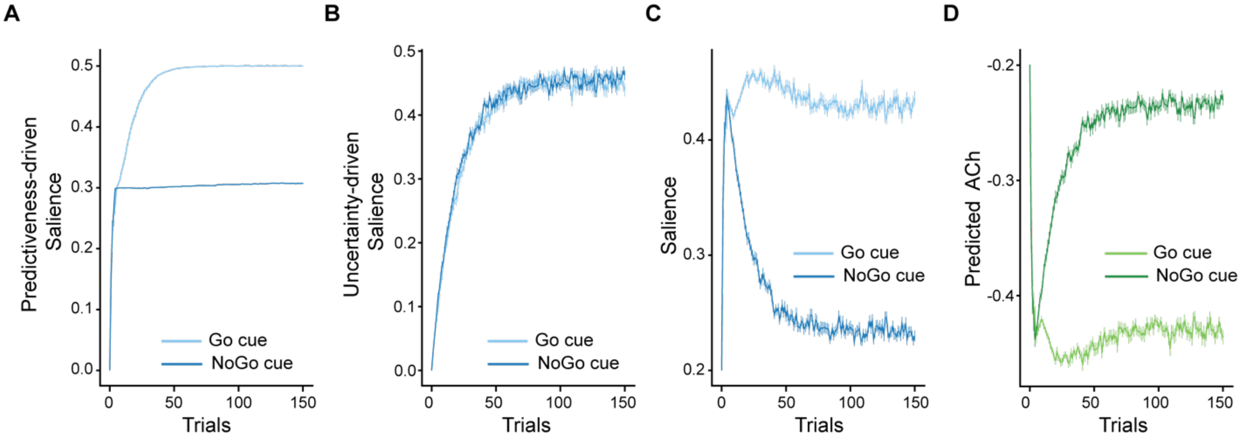

Fig. S5. Esber and Haselgrove's model explains the ACh responses during the Go/NoGo task (Experiment 1). (**A**) The predictiveness-driven salience (PDS) component predicted by the E-H model. (**B**) The uncertainty-driven salience (UDS) component predicted by the E-H model. (**C**) The net salience calculated as the difference between PDS and UDS. (**D**) ACh responses predicted by the E-H model. The error bars indicate S.E.M. across runs.

Fig. S6.

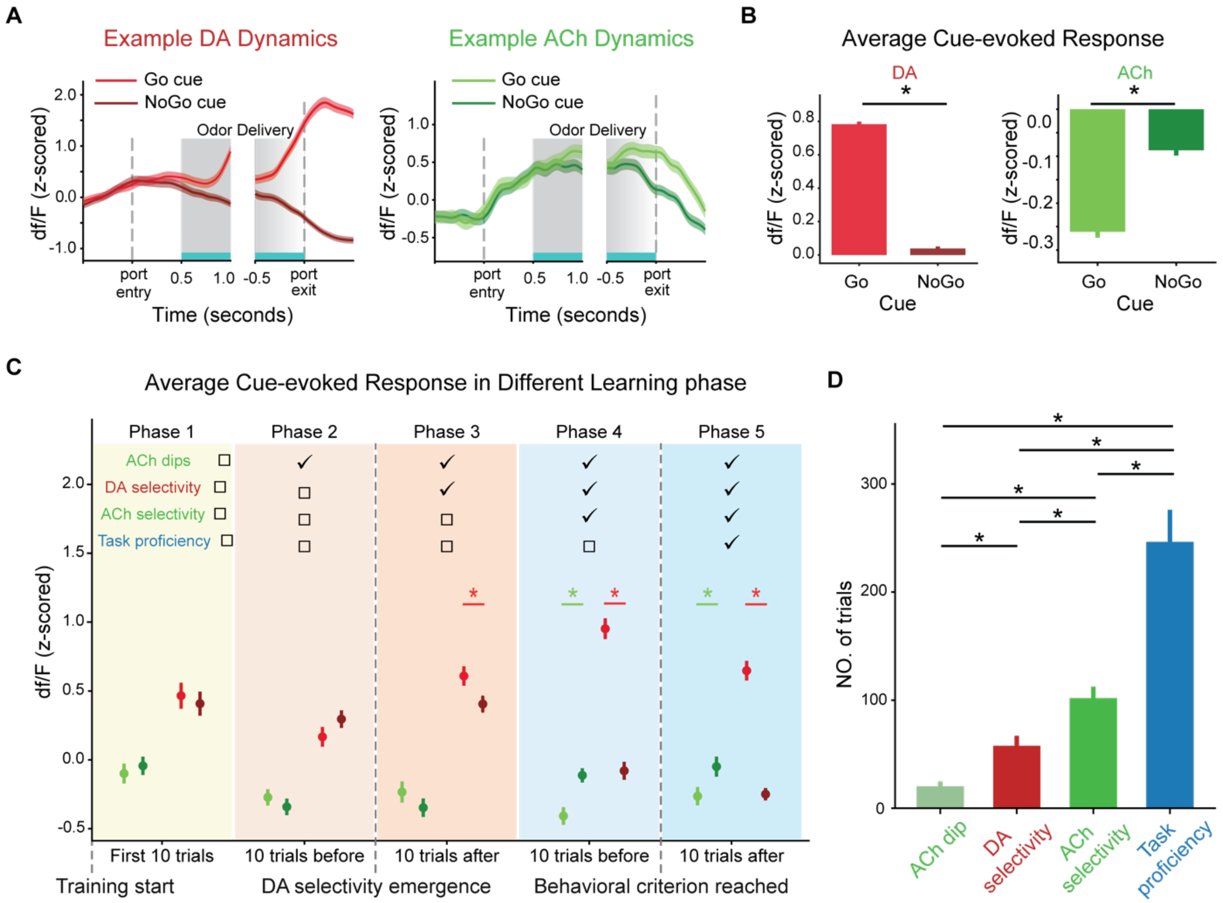

Fig. S6. Dynamics of DA and ACh during the Go/NoGo task (Experiment 1) including the outlier rat. (A). The DA (left panel) and ACh (right panel) dynamics within trials from the outlier rat. **(B-D)** Analyses as presented in fig. 2, now including the outlier rat.
