# Supplementary material for: Accumbal acetylcholine signals associative salience
Source: bioRxiv. 2025 Jan 6:2025.01.06.631529. Preprint. [Version 1] doi: 10.1101/2025.01.06.631529 (PMC11741319; doi:10.1101/2025.01.06.631529)
Supplement: Supplementary file 1 [file media-1.docx]

Document S1 

**The attentional associative learning models based on Esber and Haselgrove (2011)**

Esber and Haselgrove (2011) is another influential hybrid attentional associative learning model (hereafter referred to as the E-H model). Here, we adopted the E-H model to reproduce the ACh responses we observed in the Go/No-Go task. The Go/No-GO task structure was formalized as we did for the other models in the main text.

Like the models discussed in the main text, the E-H model represents a conditioned stimulus's associative strength through two components: an excitatory component for positive reinforcer associations and an inhibitory component for negative reinforcer associations. The associative updating mechanism follows similar principles to the previously discussed models, a distinction lies in its updating pattern. The E-H model updates both positive and negative associations on every trial, rather than selectively updating positive associations only during trials with positive RPE and negative associations only during trials with negative RPE.

Similar to Model 3, the E-H model incorporates both predictiveness-driven and uncertainty-driven components in calculating salience. However, these two salience components are defined differently from previous models. In the E-H model, the predictive-drive salience, $α$, is defined as the sum of positive and negative association strength and we updated $α$ in a temporal difference manner. This follows from the assumption that the cue will signal emotionally potent positive and negative reinforcers simultaneously, which have additive effects on salience:

$δ_{α}=V_{A}+{\bar{V}}_{A}-α$

$α=(1-η_{α})⋅α+η_{α}⋅δ_{α}+N(0, 0.05)$

Unlike Le Pelly’s model, which focuses on outcome prediction uncertainty, the E-H model shifts the focus from outcome uncertainty to cue uncertainty, i.e., the uncertainty of the cue representation based on its context and preceding cues. Thus, the uncertainty-driven salience, $σ$, decreases as the cue becomes more predictable from contextual and preceding events.

$δ_{α}=p -σ$ 

$σ=(1-η_{σ})⋅σ+η_{σ}⋅δ_{α}+N(0, 0.05)$

where $p=1$ when cue A is represented in the current trial. Otherwise, $p=0$. 

The E-H model determines a cue's overall salience, $S$, by calculating the difference between its predictiveness-driven and uncertainty-driven components, rather than their sum. This approach aligns with the model's assumption that a cue becomes less salient when it becomes predictable.

$S=b+α-ω⋅σ$

where $ω=0.6$ determines the relative contribution of $α$ and $σ$. $b=0.2$ is the baseline for the salience. When we ran this model, $η_{V}=0.4$, $η_{α}=0.5$, and $η_{σ}=0.05$.

**Figure S1**. Histological verification and photometry recording locations. **a.** Representative histological microphotograph with histological detection of both sensors. **b.** Location of fiber tips in the NAcc for all recorded rats (N=23 rats). Among these, 5 rats had fibers implanted in both hemispheres and 18 rats had fibers implanted in a one hemisphere.

**Figure S2. Determination of VAR lag length and Predict cue-evoked ACh response with DA responses. a.** Bayesian Information Criterion (BIC) values for VAR models predicting cue value updates (from Figure 2e) across different lag lengths. The circle indicate the lag ($N_{lag}$ =1) with lowest BIC value. **b.** BIC values for VAR models predicting cue-evoked ACh signals based on reward-evoked DA signals, their absolute values, and past cue-evoked ACh responses, with varying lag lengths. The circle indicate the lag ($N_{lag}$ =1) with lowest BIC value. **c.** F-values from the Granger causality test for the actual data (dark grey bars) versus shuffle data (light grey bars). No significant effects were found for reward-evoked DA signals (p-value=0.66) or their absolute value (p-value=0.45) on the subsequent cue-evoked ACh signal. The error bars in indicate S.E across trials.

**Figure S3. Dynamics of DA and ACh during the GO/NO-GO task (Experiment 1) including the outlier rat.** **a.** The DA (left panel) and ACh (right panel) dynamics within trials from the outlier rat. **(b-e)** Analyses as presented in Figure 2, now including the outlier rat. 

**Figure S4. Esber and Haselgrove’s model explains the ACh responses during the GO/NO-GO task (Experiment 1).** **a.** The predictiveness-driven salience (PDS) component predicted by the E-H model. **b.** The uncertainty-driven salience (UDS) component predicted by the E-H model. **c.** The net salience calculated as the difference between PDS and UDS. **d.** ACh responses predicted by the E-H model. The error bars indicate S.E.M. across runs.
